# Comparing Life-Cycle Emissions of Biofuels for Marine Applications: Hydrothermal Liquefaction of Wet Wastes, Pyrolysis of Wood, Fischer–Tropsch Synthesis of Landfill Gas, and Solvolysis of Wood

**DOI:** 10.1021/acs.est.3c00388

**Published:** 2023-08-17

**Authors:** Farhad H. Masum, George G. Zaimes, Eric C.D. Tan, Shuyun Li, Abhijit Dutta, Karthikeyan K. Ramasamy, Troy R. Hawkins

**Affiliations:** † Argonne National Laboratory, 9700 Cass Avenue, Lemont, Illinois 60439, United States; ‡ National Renewable Energy Laboratory, Golden, Colorado 80401, United States; § Pacific Northwest National Laboratory, Richland, Washington 99352, United States

**Keywords:** life-cycle assessment, marine bio-oils, hydrothermal liquefaction, catalytic fast pyrolysis, waste-to-energy, marine shipping, greenhouse gas emissions, maritime transport

## Abstract

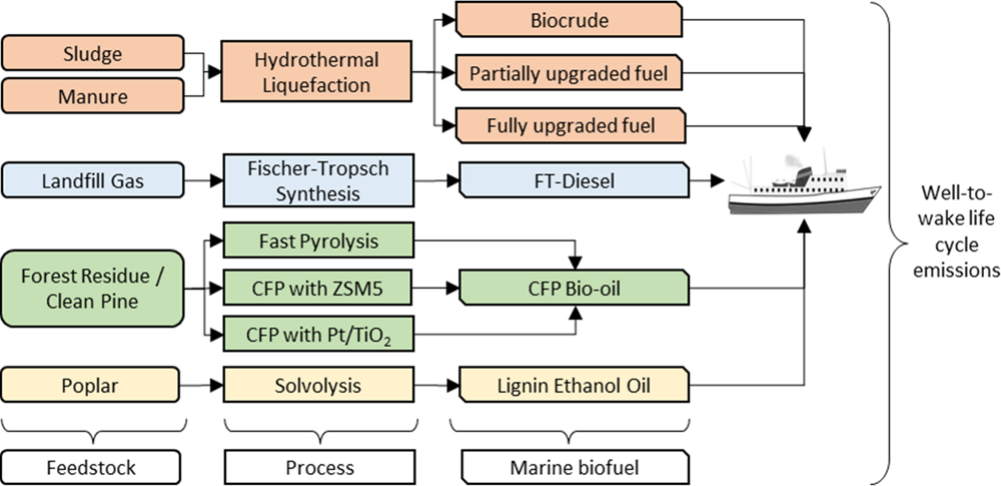

Recent restrictions on marine fuel sulfur content and a heightened regulatory focus on maritime decarbonization are driving the deployment of low-carbon and low-sulfur alternative fuels for maritime transport. In this study, we quantified the life-cycle greenhouse gas and sulfur oxide emissions of several novel marine biofuel candidates and benchmarked the results against the emissions reduction targets set by the International Maritime Organization. A total of 11 biofuel pathways via four conversion processes are considered, including (1) biocrudes derived from hydrothermal liquefaction of wastewater sludge and manure, (2) bio-oils from catalytic fast pyrolysis of woody biomass, (3) diesel via Fischer–Tropsch synthesis of landfill gas, and (4) lignin ethanol oil from reductive catalytic fractionation of poplar. Our analysis reveals that marine biofuels’ life-cycle greenhouse gas emissions range from −60 to 56 gCO_2_e MJ^–1^, representing a 41–163% reduction compared with conventional low-sulfur fuel oil, thus demonstrating a considerable potential for decarbonizing the maritime sector. Due to the net-negative carbon emissions from their life cycles, all waste-based pathways showed over 100% greenhouse gas reduction potential with respect to low-sulfur fuel oil. However, while most biofuel feedstocks have a naturally occurring low-sulfur content, the waste feedstocks considered here have higher sulfur content, requiring hydrotreating prior to use as a marine fuel. Combining the break-even price estimates from a published techno-economic analysis, which was performed concurrently with this study, the marginal greenhouse gas abatement cost was estimated to range from −$120 to $370 tCO_2_e^–1^ across the pathways considered. Lower marginal greenhouse gas abatement costs were associated with waste-based pathways, while higher marginal greenhouse gas abatement costs were associated with the other biomass-based pathways. Except for lignin ethanol oil, all candidates show the potential to be competitive with a carbon credit of $200 tCO_2_e^–1^ in 2016 dollars, which is within the range of prices recently received in connection with California’s low-carbon fuel standard.

## Abstract 2

Biofuels could replace conventional fossil marine fuel to reduce life-cycle greenhouse gas emissions from the shipping industry and could be made competitive with fossil fuels with recently available compliance credits.

## 1 Introduction

Reducing greenhouse gas (GHG) emissions has gained considerable attention in recent years as GHG-induced global warming threatens catastrophic changes to Earth’s climate. One significant source of GHGs is the maritime shipping sector, which consumes about 5 million barrels of oil per day and is responsible for approximately 3% of all anthropogenic GHG emissions.^1−3^ This translates to over 1 billion metric tons of carbon dioxide equivalents emitted into the atmosphere and reflects a 9.6% increase in emissions compared to 2012 levels.^1,3−5^ The increasing trend in global shipping projects an increase in these emissions of 50–250% by 2050.^6^ This is consistent with the projection that marine fuel consumption is expected to increase by about 44% by 2050.^7^ Besides GHG emissions, marine transport accounted for 13 and 15% of global SO_X_ and NO_X_ emissions (about 11.3 and 20.9 million t), respectively.^6,8^ These emissions contribute to ocean acidification and pose severe threats to the environment and human health.^9−15^

To combat these adverse impacts, the International Maritime Organization (IMO), which governs international shipping, set a target of a 50% reduction in GHG emissions compared to the 2008 level in this sector by 2050.^16^ IMO also expressed a goal for maritime shipping to become carbon neutral by 2100. Additionally, IMO placed a stricter 0.5% limit on fuel sulfur content, which is expected to reduce SO_X_ emissions by 77%.^17,18^ With these restrictions, the primary objective was to replace and/or reduce the consumption of sulfur-rich heavy fuel oil (HFO; 1% sulfur) or residual fuel oil. HFO, which is the leftover heavier fractions from the petroleum refining process, has been the predominant marine fuel option since the 1960s. HFO is a low-quality fuel, produced essentially as a byproduct of the refining process, and is more emissions-intensive than other commonly used fuels. However, its widespread consumption is attributed to its low cost, availability, and existing infrastructure. IMO compliant very low-sulfur fuel oil (LSFO) with 0.5% sulfur costs about $646 per tonne^19^ and emits about 96 g CO_2_e MJ^–1^ of energy in the US.^20^ However, in a few published studies, it is reported to emit higher GHG estimates, between 160 and 200 g CO_2_e MJ^–1^ of energy.^21−23^

The marine shipping sector is economically important to global trade. Maritime trade moves more than 80% of global trade by volume and 70% by value, making it vital to the global economy.^24,25^ The abovementioned restrictions on marine fuel sulfur content and a heightened regulatory focus on maritime decarbonization are reshaping this critical sector’s energy landscape and driving the deployment of low-carbon, low-sulfur, and cost-competitive alternative fuels for maritime transport.^26−28^ These regulations present a substantial challenge for ship operators, as maritime shipping often has thin profit margins.

Emission reduction can potentially be achieved by improving efficiency by altering ships’ hull designs or improving operations, such as speed optimization or capacity utilization.^29^ In addition to these efficiency gains, biofuels are a near-term option that could be used as a blend in the existing fleet or in new or retrofitted ships with fuel handling systems suitable for biofuels. Biofuels are a promising liquid energy carrier for maritime transport due to their low-sulfur and low-carbon intensity, high energy density, and potential compatibility with existing marine engines and fuel infrastructure.^30,31^ However, marine biofuels are usually more expensive than the LSFO and require subsidies to make them price competitive with their fossil counterparts.^32−35^ Since fuel accounts for the majority of the operating costs,^36^ any fuel replacing LSFO must be price competitive.

From that perspective, biofuels created from organic waste materials could offer a low-cost alternative to LSFO, while further lowering carbon intensity through offsetting emissions associated with conventional management practices. Waste feedstocks, such as manure and sludge, can be collected at a lower cost and converted to produce marine biofuel. In a Dutch case study, marine biofuel produced from hydrothermal liquefaction (HTL) of sewage sludge provided at least three times lower GHG emissions compared to the business-as-usual (BAU) scenario.^37^ Additionally, fast pyrolysis (FP) bio-oil, produced from waste woody feedstock, such as logging residue, was found to be suitable to blend with LSFO, in terms of good blend stability, polymerization inhibition, and reduced viscosity.^38^ FP bio-oil offers considerable storage and transport advantages and is a potential source of several more valuable chemicals than fuels. There is also an interest in producing liquefied biogas and lignin ethanol oil (LEO) for their potential for sustainable shipping in terms of reduced hydrocarbon and NO_X_ emissions.^39−42^ The major argument for these fuels lies in the minimal processing required to blend them with LSFO or drop them into existing marine tech with no retrofit cost.^43^ These fuels also produce coproducts, such as electricity, acetone, MEK (methyl-ethyl ketone), and cellulosic ethanol, which add coproduct credit and further advance their cost competitiveness.^44,45^

With that background, our goal was to evaluate the life-cycle environmental impacts of low-cost biofuel pathways via four major conversion methods: —HTL of manure and sludge, catalytic fast pyrolysis (CFP) of woody biomass, Fischer–Tropsch diesel (FTD) of landfill gas (LFG), and solvolysis of poplar biomass to produce LEO—exploring options for feedstocks, catalysts, and degrees of upgrading. Our objectives were to develop a comparable system boundary for these biofuels, estimate their GHG, SO_X_, and particulate matter (PM) emissions, compare their emissions with their fossil-fuel counterparts, and estimate the marginal GHG abatement costs (MAC) necessary to make these fuels price competitive with HFO.

While the HTL pathways were previously analyzed, this study is the first life-cycle assessment (LCA) of the HTL of biocrude pathways at different levels of upgrading for use in marine engines. This study is also a first in analyzing the LCA of CFP and LEO pathways of woody biomass for marine fuel. FTD of LFG has also never been analyzed for the LCA of marine fuel. These fuels were selected because they can be produced at a reasonable cost, have the potential to be produced at an industrial scale, and provide GHG reductions,^46^ and the feedstock for these fuels does not compete with food or cause significant land-use changes. Additionally, these fuels have been the focus of the US Department of Energy’s process scale-up research and development.

This study extends our understanding of the potential role of waste-to-energy pathways in the maritime shipping industry and helps us understand the carbon intensity of these bio-oils for marine applications. It also helps inform which biofuel pathways offer the lowest marginal cost of achieving GHG objectives. Finally, this study provides insights to inform investment and research and development decisions toward a cleaner future for marine transportation.

## 2 Methods

### 2.1 Goal and Scope Definition

The goal of this study is to assess the life-cycle carbon intensities and marginal cost of GHG abatement of potential low-cost biofuel substitutes for LSFO and to screen for potential tradeoffs for criteria air pollutants. The scope includes the full life cycle of each fuel pathway, including feedstock acquisition, feedstock logistics and preprocessing, conversion, fuel use, and the supply chains of all required inputs. The LCA for waste-based pathways were consequential and their counterfactual scenario or conventional waste management is discussed in the life-cycle inventory section (Sections 2.2.1 and 2.2.3). The LCA for biomass-based pathways was attributional and does not include the counterfactual scenario. The modeling is performed using Argonne National Laboratory’s Greenhouse Gases, Regulated Emissions, and Energy Use in Technologies model, 2022 version (GREET 2022).^20^ The metrics assessed include global warming potential, calculated using the IPCC AR5 (Intergovernmental Panel on Climate Change: Assessment Report) 100-year characterization factors; criteria air pollutant emissions, as defined by the GREET model; and marginal cost of GHG abatement (USD/gCO_2_-eq.). The functional unit for this study was 1 MJ of marine fuel.

### 2.2 Life-Cycle Inventory of Biofuel Pathways

**Figure 1:**
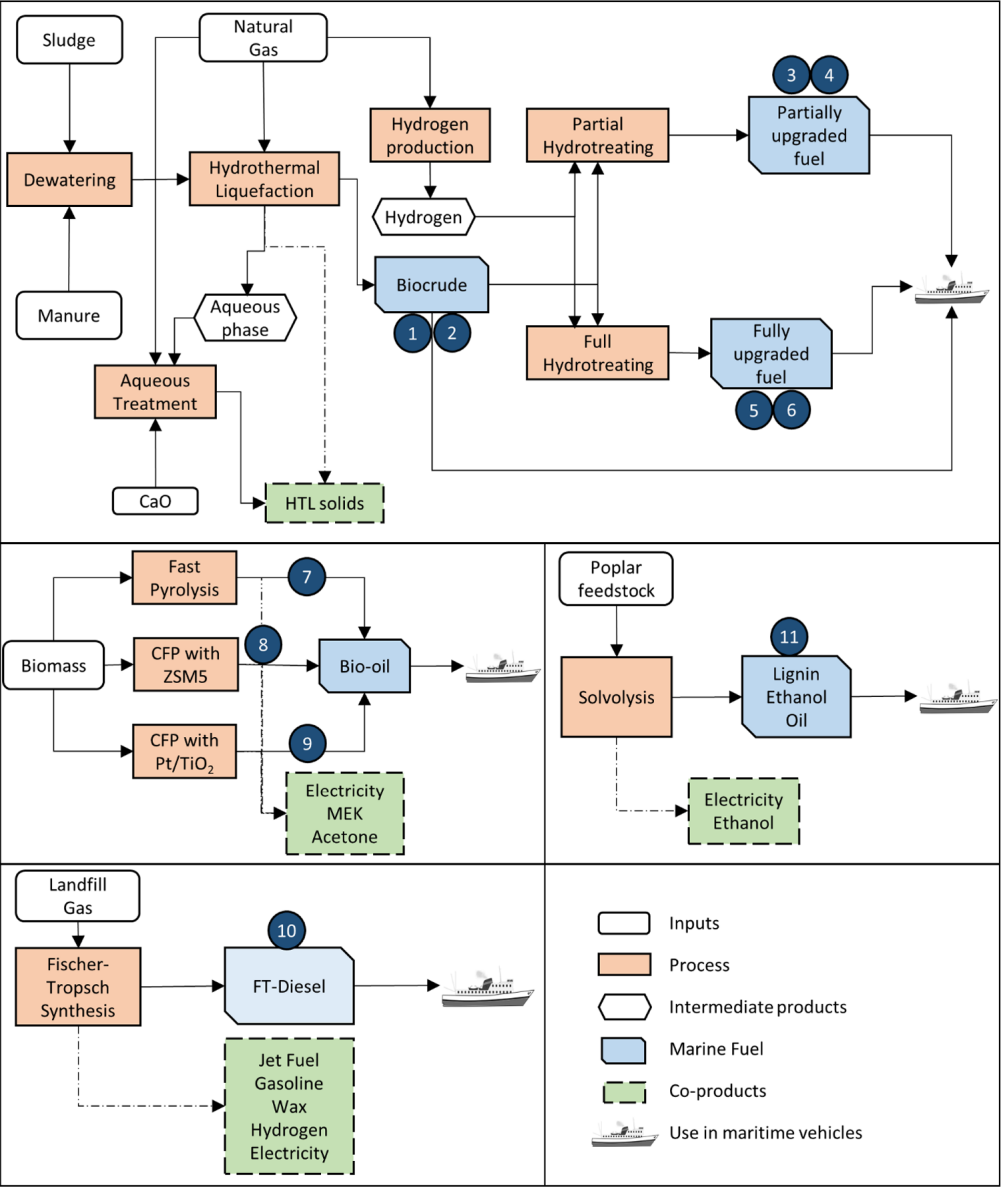
System boundary of biofuel candidates. HTL = hydrothermal liquefaction, CFP = Catalytic fast pyrolysis, FT = Fischer–Tropsch. 1 = biocrude from sludge, 2 = biocrude from manure, 3 = partially upgraded fuel from sludge, 4 = partially upgraded fuel from manure, 5 = fully upgraded fuel from sludge, 6 = fully upgraded fuel from manure, 7 = bio-oil from fast pyrolysis, 8 = bio-oil from CFP with Zeolite Socony Mobil–5, 9 = bio-oil from CFP with platinum/titanium dioxide, 10 = FT diesel, 11 = lignin ethanol oil.

We analyzed 11 distinct pathways, including five different feedstocks and four major conversion processes (Figure 1). Key characteristics of the pathways are reported in Table 1. Further details regarding the process design and techno-economic analysis of these pathways can also be found in a companion article.^46^ All of these biofuels were compared with LSFO with 0.5% sulfur for GHG and other criteria air pollutants.

**Table 1:** Key Characteristics of the Conversion Pathways and Counterfactual Scenarios

feedstock	conversion & upgrading	primary fuel	coproducts	energy allocation (%)	counterfactual	LCA type
sludge	hydrothermal liquefaction	biocrude	N/A	100	conventional sludge waste management	consequential
	hydrothermal liquefaction & partial hydrotreating	partially upgraded fuel	N/A	100		
	hydrothermal liquefaction & full hydrotreating	fully upgraded fuel	N/A	100		
manure	hydrothermal liquefaction	biocrude	N/A	100	Conventional manure waste management	
	hydrothermal liquefaction & partial hydrotreating	partially upgraded fuel	N/A	100		
	hydrothermal liquefaction & full hydrotreating	fully upgraded fuel	N/A	100		
Woody biomass	fast pyrolysis (FP) & hydrotreating	FP bio-oil	electricity	98	N/A	attributional
	catalytic fast pyrolysis (CFP) [ZSM5] & hydrotreating	CFP bio-oil	electricity, acetone	78	N/A	
	CFP [Pt/TiO2] & hydrotreating	CFP bio-oil	electricity, acetone, methyl-ethyl ketone	81	N/A	
landfill gas (LFG)	Fischer–Tropsch (FT) synthesis	FT diesel	FT-jet, FT-gasoline, wax, hydrogen, electricity	16	LFG flaring	consequential
poplar	lignin solvolysis	lignin ethanol oil	cellulosic ethanol	48	N/A	attributional

#### 2.2.1 Hydrothermal Liquefaction

We analyzed two pathways for producing biocrude via HTL of sludge and manure. The HTL plant capacity was one metric ton per day, with sludge and manure collection radii modeled to be 47.3 and 115.1 km, respectively. We considered three levels of hydrotreating the biocrude from each feedstock: no treatment, partial treatment, or full treatment, with the hydrogen considered to be produced from natural gas via steam methane reforming. Therefore, six HTL pathways were analyzed: untreated biocrude from sludge, untreated biocrude from manure, partially hydrotreated fuel from sludge, partially hydrotreated fuel from manure, fully hydrotreated fuel from sludge, and fully hydrotreated fuel from manure. Biosolids are produced as a byproduct of the HTL process and the treatment of aqueous waste. These biosolids are assumed to be land applied with a carbon sequestration credit calculated based on the estimated fraction of decomposition (20%) versus long-term carbon additions (80%) to the soil.^47^ Conventional waste management was regarded as the counterfactual scenario for the sludge and manure pathways. In conventional waste management, 19.1 g CH_4_ kg^–1^ of sludge and 10.5 g CH_4_ kg^–1^ of manure are emitted into the atmosphere. These methane emissions were avoided when the waste feedstocks were diverted for energy generation. Conventional manure management produces organic fertilizers (13.43 kg N t^–1^, 7.96 kg P t^–1^, and 3.98 kg K t^–1^ of wet manure).^47^ When manure was rerouted to generate marine fuel, the counterfactual scenario included the production of inorganic fertilizers farmers were required to use in the absence of those organic fertilizers. Wet waste properties, biocrude and fuel product properties, inputs, and outputs for biocrude and upgraded fuel production are presented in the Supporting Information (Tables S1 and S2). The energy allocation ratio for all HTL pathways was 100% since there were no energy coproducts.

#### 2.2.2 Fast Pyrolysis

Three FP pathways producing bio-oil were analyzed: FP without a catalyst, and two CFP alternatives, the first with Zeolite Socony Mobil–5 (CFP with ZSM5) and the second with platinum/titanium dioxide (CFP with Pt/TiO_2_). The feedstocks, which are blended woody biomass comprised of 50% forest residue and 50% clean pine, are assumed to be transported 167 and 82 km, respectively, to the CFP plant.

Excess electricity was produced in all three pathways. Acetone was produced in both CFP pathways, and MEK was produced in the CFP with ZSM5 pathway. We used displacement allocation for all three FP and CFP pathways, in which electricity, MEK, and acetone were displaced, therefore, receiving a displacement credit. After that, 100% of emissions were allocated to marine fuels. The detailed inventories for the three fast pyrolysis pathways are provided in the Supporting Information (Table S3).

#### 2.2.3 Fischer–Tropsch Synthesis

The Fischer–Tropsch (FT) pathway uses LFG fed into a steam reformer to make synthesis gas, which is subsequently sent to the FT reactor to produce synthetic diesel. The process uses catalysts, including tar reformer catalyst, hydro-isomerization catalyst, zinc oxide, cobalt-based FT-synthesis catalyst, and cobalt–molybdenum- or nickel–molybdenum-based hydrotreating catalyst.

Jet fuel, gasoline, hydrogen, and electricity were produced as coproducts during the process. We used a hybrid allocation method, in which electricity was displaced, and the lower heating values of other liquid fuels were used to calculate the energy allocation ratio. For the FTD pathway, the energy allocation ratio was 15.63%. A detailed inventory for the LFG-based FTD pathway, which was required to calculate the energy allocation ratio, is provided in the Supporting Information (Table S4). The lower heating values of liquid fuels were collected from the GREET database.^20^

#### 2.2.4 Lignin Solvolysis

The lignin solvolysis pathway uses lignin extracted from poplar feedstock to make LEO. The biomass feedstock first undergoes reductive catalytic fractionation (RCF). The integrated biorefinery produces both ethanol and a depolymerized lignin-rich oil and allows for the integration of lignin–ethanol solvolysis. While a portion of ethanol is degraded to CO and CO_2_ in the RCF reactor, a majority is recovered via distillation and recycled back to the reactor, resulting in a net ethanol consumption of approximately 60 g of ethanol per kg of LEO product. Ethanol produced from the biorefinery supplies the ethanol for RCF. Solvolysis used 1.45 MJ of natural gas for 1 MJ of LEO, nearly half (47.6%) of which is used to vaporize the excess ethanol solvent for LEO separation. The process produces LEO and ethanol fuel products, and excess electricity is exported to the grid as a coproduct. For allocation, electricity is displaced, and the energy allocation ratio was calculated to be 48.1%, combined with the cellulosic ethanol output (0.93 MJ MJ^–1^ of LEO). A detailed inventory for the LEO pathway is provided in the Supporting Information (Table S5).

### 2.3 Life-Cycle Emissions

We used the Excel version of the GREET 2022 model to estimate the life-cycle GHG emissions and criteria air pollutant emissions.^20^ GREET is an LCA tool developed and updated annually by Argonne National Laboratory.^48^

We considered a hybrid allocation for conducting the LCA, explained by the following equation:
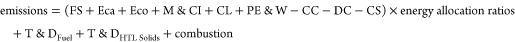
1

Emission factors for feedstock production (FS), energy carriers (Eca) and combustion (Eco), material and chemical inputs (M&CI), catalysts(CL), and process emissions and water use (PE&W) are presented in Supporting Information (Tables S6–S10). Regarding counterfactual credit(CC), as mentioned in Section 2.2.1, conventional sludge and manure management was considered as the counterfactual scenario for HTL pathway’s sludge and manure feedstock, respectively. The counterfactual credit for the manure feedstock was larger than the sludge pathway since diverting manure from the conventional management system to produce marine biofuel avoids the emissions associated with conventional manure management (e.g., in open pits or lagoons without methane capture/flaring). The manure used for the HTL process, rather than as a fertilizer, was considered to be replaced by inorganic fertilizers, and the emissions from the use of inorganic fertilizers were accounted for in the counterfactual credit calculation. LFG flaring was assumed to be the counterfactual scenario for the FTD pathway. Emission parameters for counterfactual credit and displacement credit (DC) for displaced electricity, acetone, and MEK are presented in Table S11. CS refers to the carbon sequestration from HTL solids and solids from the aqueous treatment, parameters for which are available in Table S12.

T&D_Fuel_ refers to the transportation of marine fuel by truck 104 miles one-way. T&D_HTL Solids_ refers to the transportation and distribution of HTL solids and solids from the aqueous treatment. Transportation distances for sludge and manure were mentioned in Section 2.2.1. Combustion refers to the emissions from combusting fuel. Biogenic CO_2_ is not assigned a GHG impact as an equivalent amount of CO_2_ was taken up by the biomass during plant growth. However, biogenic CH_4_ is included in the combustion emissions. We leveraged the GREET emission factor for LSFO (0.5% sulfur) to calculate our emissions for fuel combustion^20^ due to the lack of reliable data on biocrude/bio-oil emission factors. Emission parameters for T&D_Fuel_, T&D_HTL Solids_, and combustion are presented in the Supporting Information (Tables S13 and S14).

### 2.4 Marginal Abatement Cost

We calculated the marginal GHG abatement cost (MAC) by leveraging minimum fuel sale price results provided by a techno-economic analysis performed concurrently with this study.^46^ We estimated the MAC of carbon using the following formula:
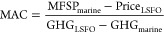
2where MFSP_marine_ was the minimum fuel selling price of marine biofuel including the coproduct credit, listed in the Supporting Information (Table S15). The Supporting Information also contains the price per gallon of LSFO equivalent, price per metric ton of LSFO equivalent, and price per gallon of gasoline equivalent. Price_LSFO_ was the price of LSFO, conventionally used in marine transportation,^19^ $0.015 MJ^–1^ or $612.5 t^–1^ of LSFO ($1.86 per gallon of gasoline equivalent, where LHV of gasoline is 122.5 MJ gallon^–1^); GHG_LSFO_ was the GHG emission from LSFO,^20^ 96 gCO_2_e MJ^–1^; and GHG_marine_ was the well-to-wake GHG emission from marine biofuel, as discussed in Section 3.1. We considered a minimum ($0.007 MJ^–1^ or $262.8 t^–1^ or $0.86 per gallon of gasoline equivalent) and maximum ($0.028 MJ^–1^ or $1136.5 t^–1^ or $3.37 per gallon of gasoline equivalent) range for Price_LSFO_ to estimate the range for MAC. This range was acquired from historical LSFO prices between January 2020 and January 2023.^19^

### 2.5 Sensitivity Analysis

We conducted a sensitivity analysis for two scenarios: (i) variation in external energy requirement (±20%), which included changes in the natural gas, electricity, and no. 2 diesel fuel amounts, and (ii) variation in fuel yield (±20%). The results are presented in Section 3.5.

## 3 Results and Discussion

### 3.1 GHG Emissions

**Figure 2:**
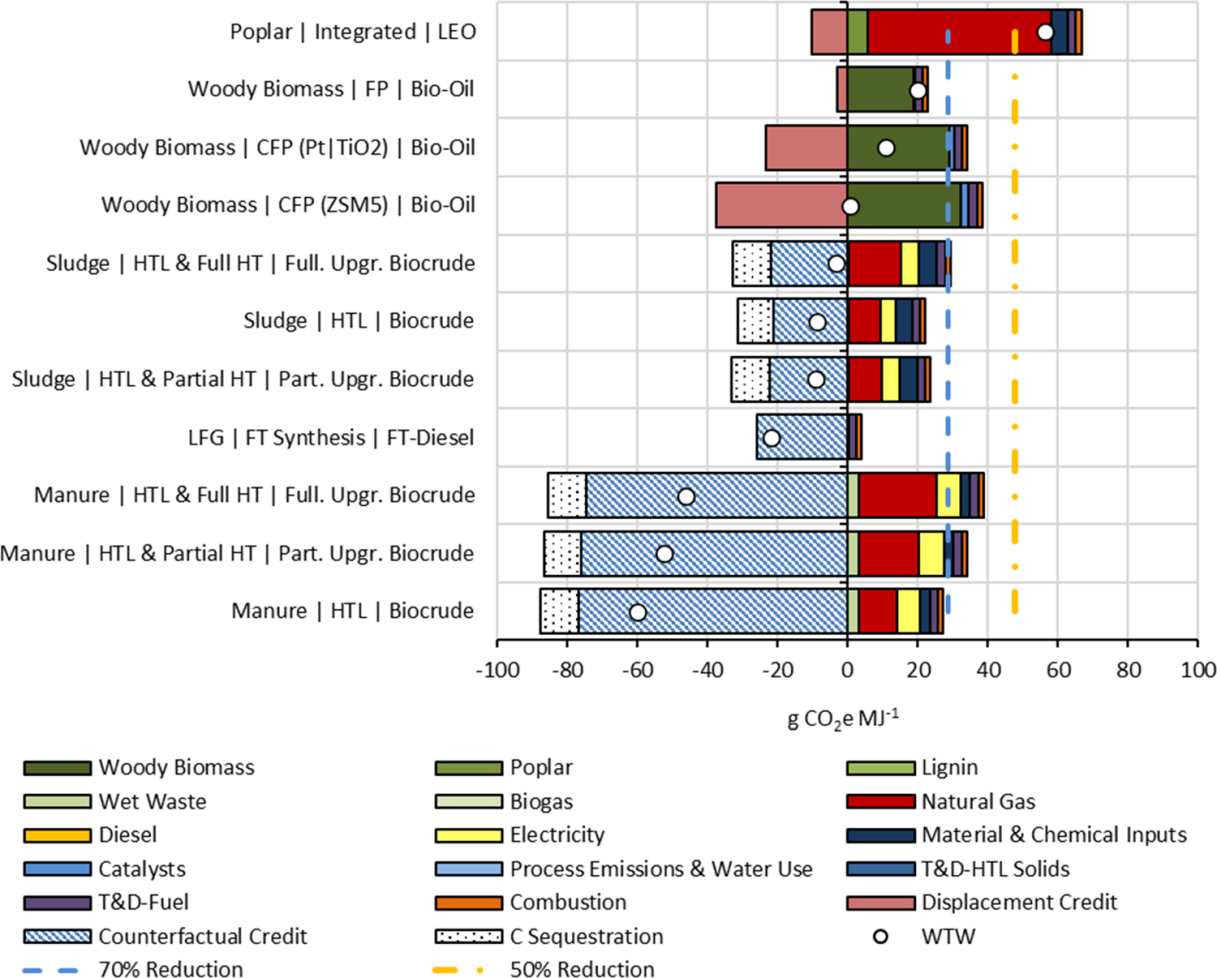
Life-cycle GHG emissions of marine biofuel pathways. Part. Upgr. and Full. Upgr. refer to partially upgraded and fully upgraded, respectively. WTW refers to total well-to-wake emissions.

Life-cycle GHG emissions ranged from −60 to 56 gCO_2_e MJ^–1^ of marine fuel (Figure 2), suggesting a 41–163% GHG reduction compared to conventional LSFO. Biocrude from manure feedstock had the lowest GHG intensity among all pathways, reflecting a 163% reduction in life-cycle GHG emissions compared to LSFO. All manure-based pathways achieved life-cycle GHG emissions reductions of 148% or greater, primarily due to the large counterfactual credit from avoiding the methane emissions from open pit or lagoons in conventional manure management. HTL of sludge also showed at least a 103% reduction in GHG emissions. It is important to mention here that while HTL pathways provide the highest GHG reduction, the total production of these fuels is subject to the availability of their primary feedstocks—sludge and manure. In the US, the total availability of these feedstocks may present a challenge in establishing necessary infrastructures due to scaling issues. In other words, to make any HTL biorefinery economically competitive, it will require a substantial quantity of these feedstocks, which may be challenging due to the scarcity of feedstock. In comparison, FP/CFP pathways based on wood and other cellulosic feedstocks have higher scale-up potential than HTL pathways assuming the system for collection and delivery of wood and wood residues could be economically scaled. For example, in the US, while 6570 t of wood was annually available at $40 t^–1^, only 443 t of wastewater sludge and 909 t of manure were available at the same price.^49,50^ The techno-economic analysis of these pathways,^46^ concurrently performed with this study, was conducted for a large biorefinery, and the feedstock prices were also assumed for a scaled-up infrastructure. However, there is a need to revisit the assumptions used in this study based on scaled-up operations in practice.

LEO from poplar feedstock was the most GHG intensive biofuel, but it still offered a 41% GHG savings compared to LSFO. Approximately 78% of all emissions in the LEO pathway were attributed to natural gas. As mentioned previously, the LEO pathway was a natural gas-intensive process. One way to improve energy or natural gas consumption is to lower the ethanol feed requirement.

Marine biofuels produced from FP of biomass provided 79–99% GHG savings. This finding is in accordance with existing studies. Tan et al.^30^ found that marine fuel from biomass provided 67–93% GHG savings compared to LSFO. Other studies^29,43,44^ showed that biofuels can provide a greater than 50% GHG reduction in a similar system boundary.

Bio-oils from FP and CFP pathways may be the most readily adoptable marine biofuels when scaling issues are considered since biomass is the most abundant feedstock among the feedstock categories considered in our study. However, since these bio-oils rely on different feedstocks and do not compete with one another from the best use of feedstock perspective, the marine fuel market could benefit from absorbing fuels produced via all these pathways. Additionally, these bio-oils can be blended as a strategy for reducing GHG emissions, while causing minimal to no disruption in current infrastructure, engine design, supply chain operations, etc.

### 3.2 SOx Emissions

**Figure 3:**
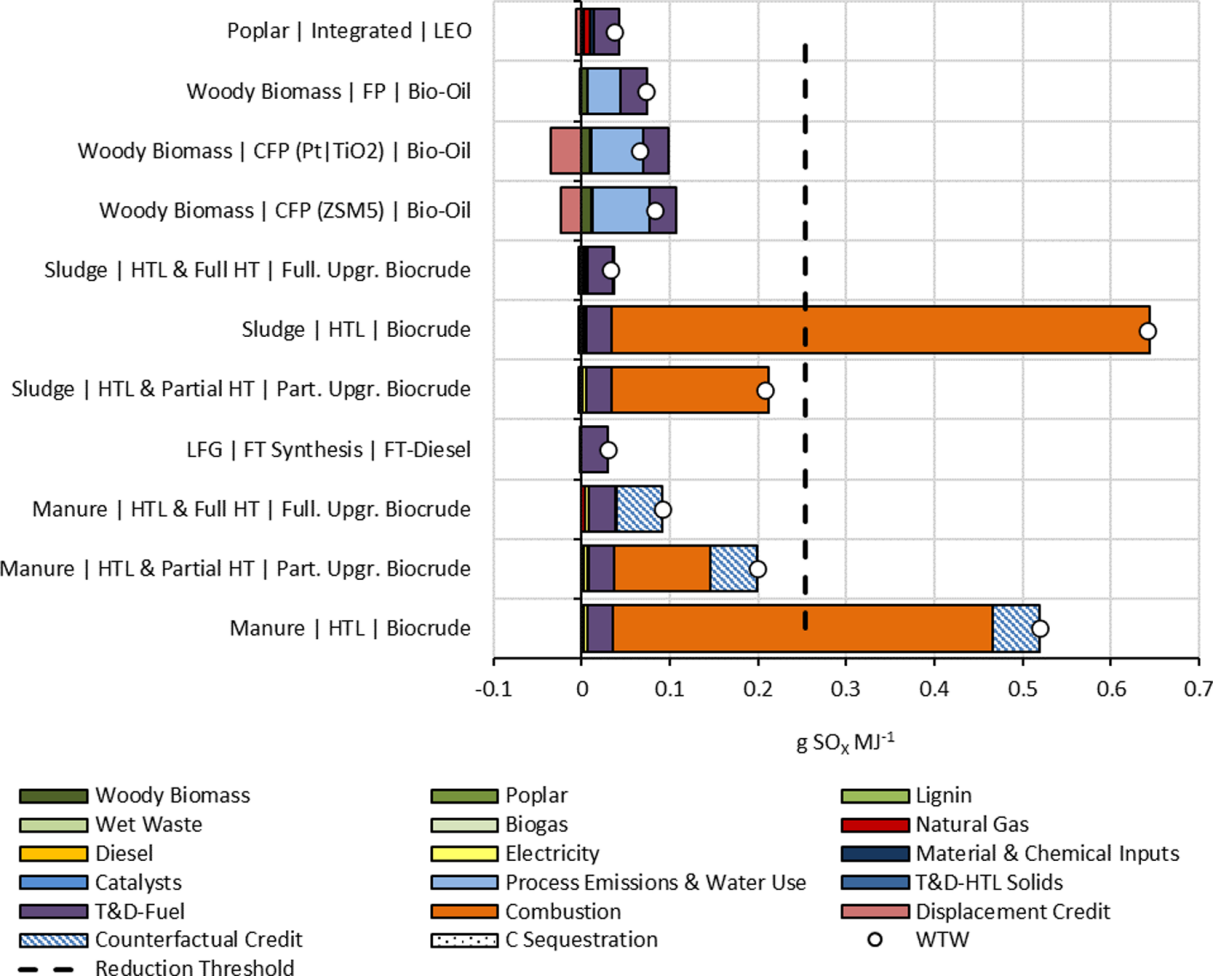
Life-cycle SOx emissions of marine biofuel pathways. Part. Upgr. and Full. Upgr. refer to partially upgraded and fully upgraded, respectively. WTW refers to total well-to-wake emissions.

Life-cycle SO_X_ emissions ranged from 0.03 to 0.64 g SO_X_ MJ^–1^ for the evaluated pathways (Figure 3). Except for untreated biocrudes, all the biofuel pathways showed lower sulfur content than the allowable limit due to the low-sulfur content of their feedstocks.

Manure- and sludge-based biocrudes that are not hydrotreated have sulfur content above the LSFO 0.5% sulfur. Therefore, HTL biocrudes would need to be hydrotreated to comply with IMO sulfur standards prior to use. The sludge-based HTL pathway can provide an 87% GHG reduction with full hydrotreating, while manure-based HTL can provide a reduction of up to 64%. However, partially hydrotreated fuel can provide 18% (sludge) and 22% (manure) relative SO_X_ savings compared to LSFO. This emphasizes the necessity of hydrotreating biocrude from sludge and manure. Combustion was the primary source of SO_X_ emissions for these pathways, constituting approximately 86 and 83% of all emissions for sludge and manure options, respectively.

For the woody biomass pathways, process emissions, and water use was the most SO_X_-intensive stage, constituting 53–91% of all emissions. The T&D stage accounted for the highest SO_X_ emissions for the LEO pathway (approximately 81%), while almost 100% of emissions for FTD from LFG came from T&D. FTD provided the highest relative SO_X_ savings (89%). Our estimates and relative SO_X_ savings were comparable to other studies.^43,51^

### 3.3 PM Emissions

**Figure 4:**
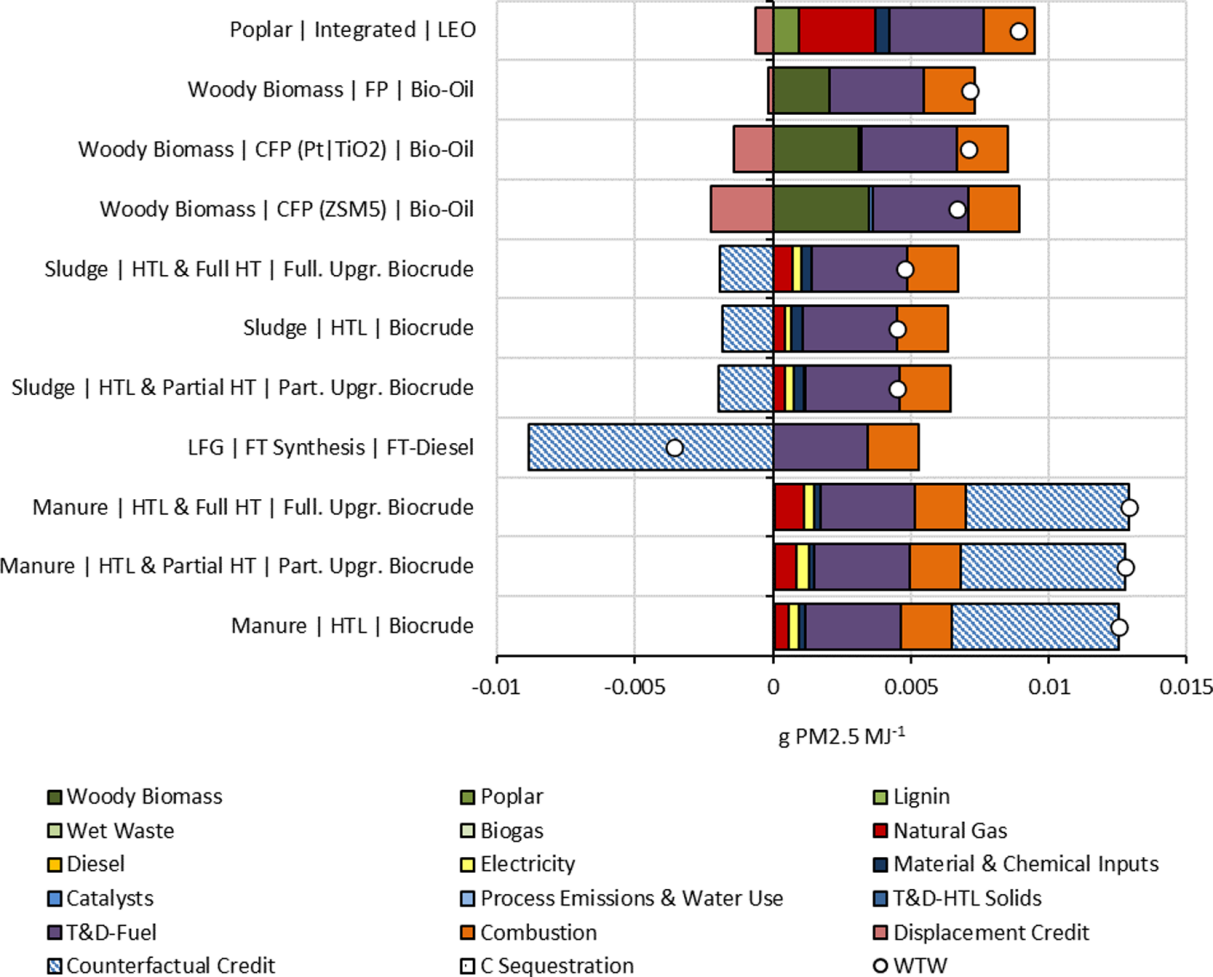
Life-cycle PM2.5 emissions of marine biofuel pathways. Part. Upgr. and Full. Upgr. refer to partially upgraded and fully upgraded, respectively. WTW refers to total well-to-wake emissions.

All marine biofuels were compliant with the existing PM emissions requirement, <0.08 g PM2.5 MJ^–1^, and provided at least an 84% reduction compared to LSFO (Figure 4). FTD from LFG received the highest counterfactual credit by avoiding PM emissions from LFG flaring and provided a 104% reduction in the process. In the case of PM10 emissions, marine biofuels provided at least a 65% and up to an 86% reduction with manure-based partially hydrotreated fuel and the FTD pathway, respectively. Transportation and distribution constituted between 27 and 76% of all PM emissions among the pathways. T&D share was the highest for manure-based pathways and the lowest for sludge-based pathways.

Along with PM10 emissions, NOx emissions, total energy requirement, and water consumption, results are graphically presented in the Supporting Information (Figures S1–S4).

### 3.4 Marginal Abatement Cost

**Figure 5:**
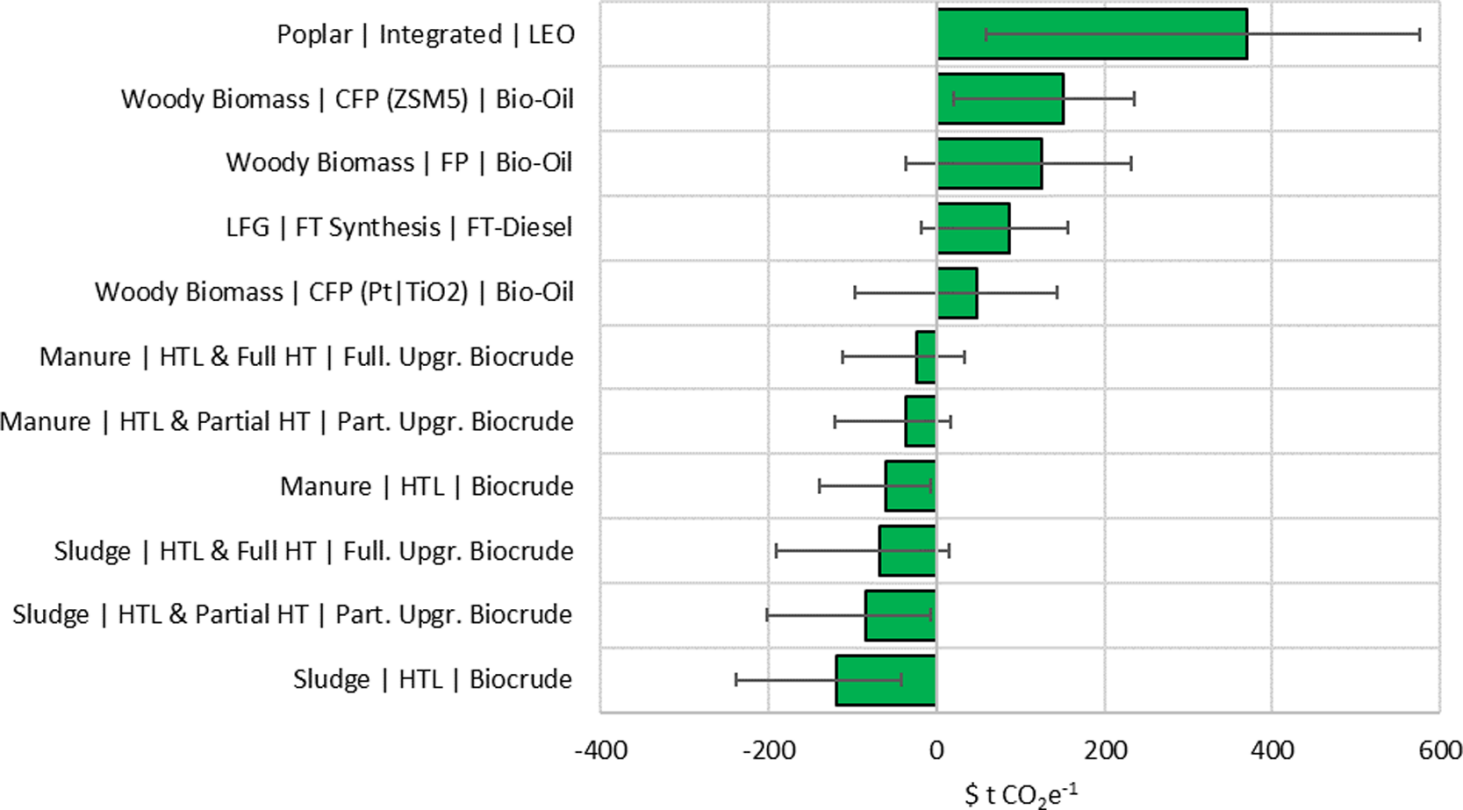
Marginal carbon abatement cost required to make biofuels competitive with conventional marine fuel oil. Part. Upgr. and Full. Upgr. refer to partially upgraded and fully upgraded, respectively.

MAC estimates ranged from -$120 to $370 tCO_2_e^–1^ across the pathways considered (Figure 5). All sludge- and manure-based fuels had a negative abatement cost, suggesting that they are already competitive with conventional LSFO and do not require any further financial incentive. Even when low LSFO cost was considered, the highest MAC required among all manure and sludge pathways was $33 tCO_2_e^–1^, which is less than the available compliance credit available from California’s low-carbon fuel standard ($200 tCO_2_e^–1^ in 2016 dollars).^52^ Bio-oil from all FP and FTD from LFG can be made competitive with less than $200 tCO_2_e^–1^ of the carbon tax on LSFO. However, with low LSFO prices, bio-oil from FP and CFP with ZSM5 cannot be price competitive, as MAC requires increases to at least $232 tCO_2_e^–1^. Bio-oil from LEO from poplar pathways was competitive only when the LSFO price was the highest within the three-year period from January 2020 to January 2023 ($1172 t^–1^). Perhaps future studies can explore non-thermal separation approaches, such as membrane separation, instead of using natural gas to vaporize the excess ethanol solvent for LEO separation.

The low MAC was due to the lower processing and upgrading required for these marine biofuels compared to other types of fuels, such as diesel or jet fuel. Ou et al.^47^ showed that mixing controlled compression ignition diesel blendstocks, produced from yellow grease, for heavy-duty diesel vehicles required between $116 and $270 tCO_2_e^–1^ of MAC. Similar to this study, they reported lower MAC for renewable diesel from swine manure HTL compared to biomass sources. Tan et al.^30^ showed that pyrolysis oil from biomass could be made competitive with less than $100 tCO_2_e^–1^ of MAC when the LSFO price is over $0.6 L^–1^. In our analysis, marine biofuels required lower MAC to be economically competitive with LSFO compared to MAC required to make sustainable aviation fuel (SAF) competitive with fossilized jet fuel. Studies have found that $206 to $420 tCO_2_e^–1^ of MAC will be required for oil seed-based SAF,^53−55^ and $234 to $263 tCO_2_e^–1^ for SAF from forest residues.^59^ High MAC, ranging between $553 and $661 tCO_2_e^–1^, were reported for synthetic fuel from hydrogen for marine purposes.^60^

Nevertheless, caution is required when interpreting results for the waste pathways because they are strongly based on assumptions regarding conventional waste management practices. Future waste management practices with reduced methane emissions may reduce the low MAC benefit for waste-based fuel pathways. Additionally, competitiveness may vary based on the variation in LCFS credit. LCFS credit ranged between $218 and $20 tCO_2_e^–1^ from August 2017 to February 2021.^56^ With the lowest LCFS credit, only the waste-based HTL pathways were competitive. The same was true when comparing these MACs with the California-Quebec carbon allowance price,^57^ $29.15 tCO_2_e^–1^. When the LSFO price was the highest, all pathways were competitive with the California-Quebec carbon allowance price, except the LEO pathway. With the lowest European Union (EU) carbon market price^58^ between January 2021 and January 2023 ($35 tCO_2_e^–1^), only HTL pathways were competitive. With the highest EU carbon market price within the same time period, CFP (Pt/TiO2) and FTD pathways become competitive along with HTL pathways. Despite being a separate market, comparing these MACs with the California-Quebec carbon allowance price or the EU carbon market price shows the international competitiveness of these fuels.

**Figure 6:**
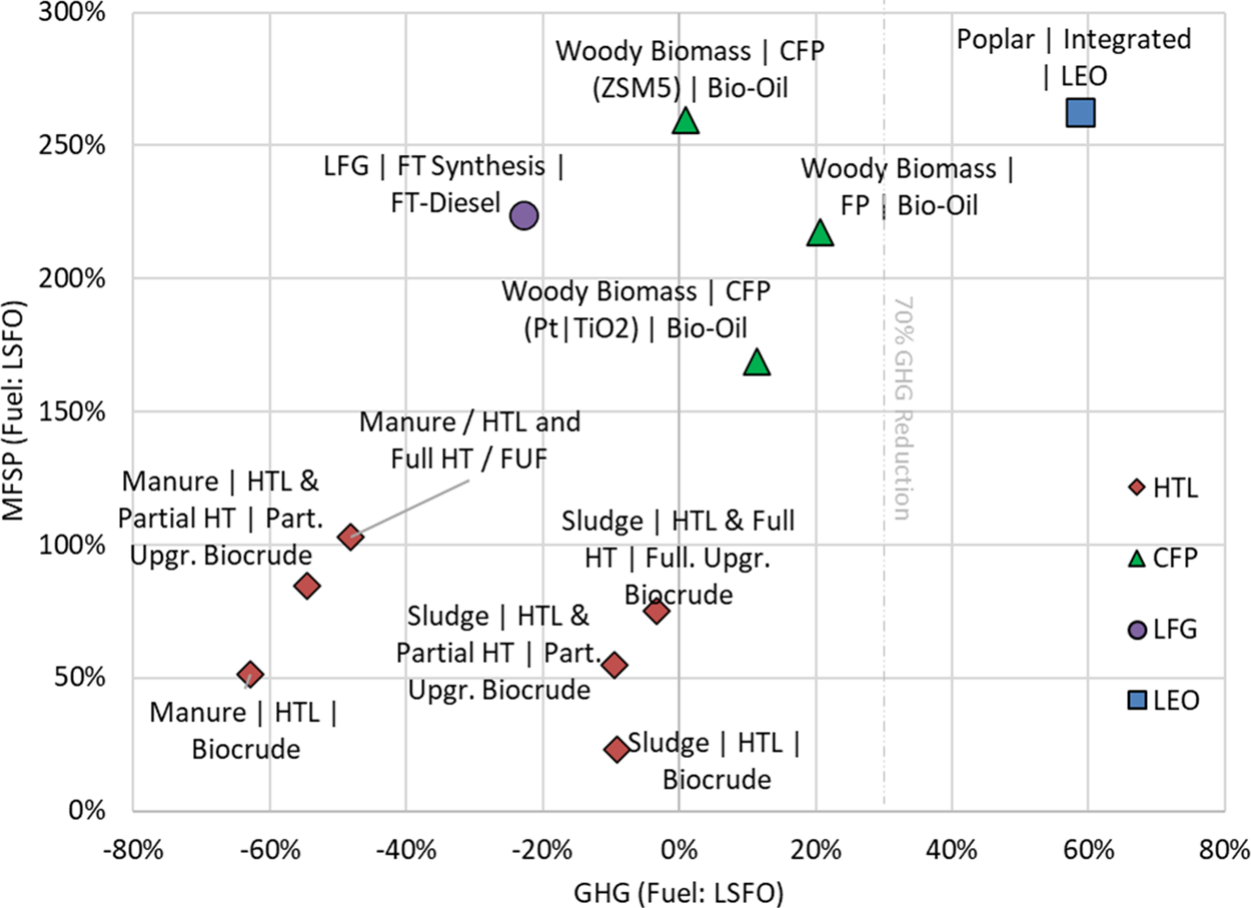
Ratio of greenhouse gas emissions and minimum fuel selling price (MFSP) with respect to conventional marine fuel oil. Part. Upgr. and Full. Upgr. refer to partially upgraded and fully upgraded, respectively.

Figure 6 shows the competitiveness of biofuels compared to the fossil fuel alternative (LSFO) for marine shipping. The bottom right cluster, consisting of manure- and sludge-based pathways, indicates more desirable economic and GHG performance as these were both cheaper and less GHG intensive than LSFO. Woody biomass-based oils are less competitive than waste-based pathways.

### 3.5 Sensitivity Analysis

**Figure 7:**
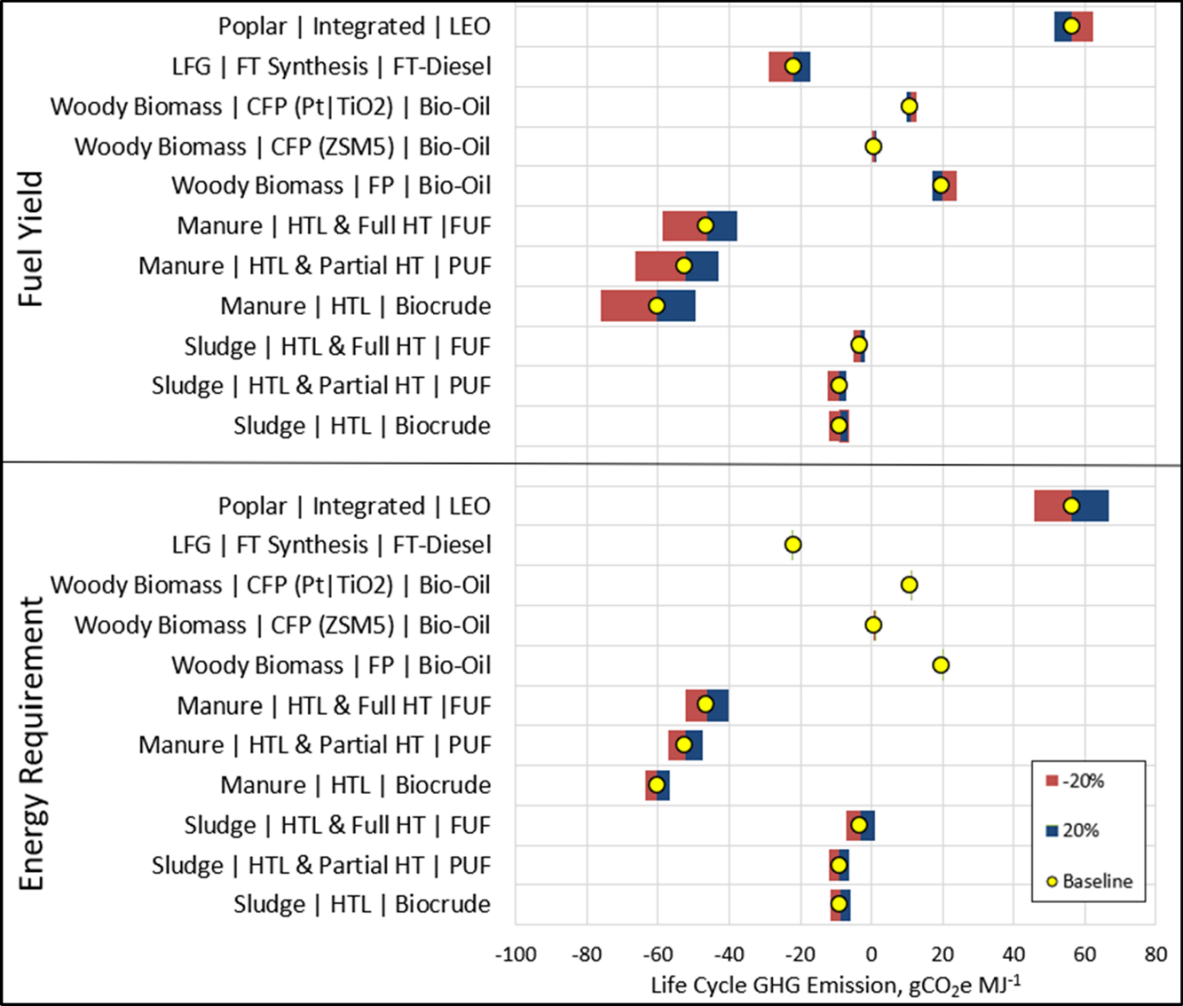
Sensitivity analysis of life-cycle carbon emissions based on varying parameters. PUF and FUF refer to partially upgraded fuel and fully upgraded fuel, respectively.

Figure 7 illustrates how changes in energy requirement and fuel yield impact the overall GHG emissions. A 20% change in the external energy requirement had the highest impact on GHG emissions from the poplar-based LEO pathway (119%), ranging from 45.9 to 66.8 g CO_2_e MJ^–1^. For FP and FT pathways, energy requirement had a negligible (less than 1%) impact since these pathways require minimal diesel fuel and no natural gas or electricity. For all pathways, emissions decreased when the external energy requirement and fuel yield were low.

While a decline in emissions with lower energy requirements makes intuitive sense, the impact of fuel yield requires further explanation. While the yield of fuel per ton of sludge or manure was low, the counterfactual credit and credits from HTL solids were unchanged, resulting in higher credit per MJ of final fuel and lower emissions. Manure-based HTL pathways had the greatest impact from variations in fuel yield. With no hydrotreating, a 20% variation in fuel yield resulted in a −183 to 122% change in GHG emissions. With full hydrotreating, emissions varied between −143 and 96%. With increased fuel yield, emissions decreased for FP, CFP with Pt/TiO2, and LEO pathways, while emissions increased for CFP with ZSM5 and FTD pathways. In the latter two pathways, since the total displaced electricity was unchanged, credit from displaced electricity per MJ of fuel decreased with increased fuel yield.

Future studies can explore other biomass sources and pathways to produce marine biofuels. For example, biodiesel from food waste and fats, oils, and grease and FT fuel from municipal solid waste^61^ can also be analyzed for cost and emissions estimates. Renewable diesel from algae is another promising feedstock that can be analyzed as a marine fuel since algal biofuel’s resource potential and its assessment as a bio-jet fuel already exist in the literature.^62,63^

## Supporting Information Available

Life-cycle inventory of fuel pathways; fuel properties and inventory; life cycle inventory for fast pyrolysis-based pathway, life cycle inventory for landfill gas-based pathway, LEO-based pathway; emission parameters for feedstock; energy carrier and energy combustion; material and chemical input; catalyst input; process emissions and water use; displacement and counterfactual credit, emission parameters for C sequestration; emission parameters for transportation; emission parameters required to replicate the study; minimum fuel selling price data; life cycle PM_10_ and NO_X_ emissions; energy and water consumption (PDF)

## Abbreviations

$: United States dollar
CFP: catalytic fast pyrolysis
CH_4_: methane
CO_2_: carbon dioxide
CO_2_e: carbon dioxide equivalent
FP: fast Pyrolysis
FT: Fischer–Tropsch
FTD: Fischer–Tropsch diesel
FUF: fully upgraded fuel
g: gram
GHG: greenhouse gas
HFO: heavy fuel oil
HTL: hydrothermal liquefaction
IMO: International Maritime Organization
LCA: life cycle assessment
LEO: lignin ethanol oil
LFG: landfill gas
LNG: liquefied natural gas
LSFO: low-sulfur fuel oil
MAC: marginal abatement cost
MEK: methyl-ethyl ketone
MFSP: minimum fuel selling price
MJ: mega joule
MTPD: metric ton per day
NO_X_: oxides of nitrogen
PM: particulate matter
PUF: partially upgraded fuel
RCF: reductive catalytic fractionation
SAF: sustainable aviation fuel
SO_X_: sulfur dioxide
t: tonne or metric ton

## References

[ref1] BP. Energy Outlook 2020 Edition: Report by British Petroleum https://www.bp.com/content/dam/bp/business-sites/en/global/corporate/pdfs/energy-economics/energy-outlook/bp-energy-outlook-2020.pdf (accessed 2022-02-07).

[ref2] ABS. Setting the Course to Low Carbon Shipping - Report by American Bureau of Shipping https://absinfo.eagle.org/acton/attachment/16130/f-982b1623-4d26-4b04-91c5-25453a6e2fba/1/-/-/-/-/low-carbon-shipping-outlook.pdf (accessed 2023-01-12).

[ref3] Faber J.; Hanayama S.; Zhang S.; Pereda P.; Comer B.; Hauerhof E.; van der Loeff W. S.; Smith T.; Zhang Y.; Kosaka H.; Adachi M.; Bonello J.-M.; Galbraith C.; Gong Z.; Hirata K.; Hummels D.; Kleijn A.; Lee D. S.; Liu Y.; Lucchesi A.; Mao X.; Muraoka E.; Osipova L.; Qian H.; Rutherford D.; de la Fuente S. S.; Yuan H.; Perico C. V.; Wu L.; Sun D.; Yoo D.-H.; Xing H. Fourth IMO Greenhouse Gas Study - Report by International Maritime Organization; London, 2020.

[ref4] Lindstad E.; Lagemann B.; Rialland A.; Gamlem G. M.; Valland A. Reduction of Maritime GHG Emissions and the Potential Role of E-Fuels. Transp. Res. D Transp. Environ. 2021, 101, 103075 10.1016/j.trd.2021.103075.

[ref5] Lindstad E.; Eskeland G. S.; Rialland A.; Valland A. Decarbonizing Maritime Transport: The Importance of Engine Technology and Regulations for LNG to Serve as a Transition Fuel. Sustainability 2020, 12, 8793 10.3390/su12218793.

[ref6] Smith T.; Jalkanen J.; Anderson B.; Corbett J.; Faber J.; Hanayama S.; O’Keeffe E.; Parker S.; Johansson L.; Aldous L.; Raucci C.; Traut M.; Ettinger S.; Nelissen D.; Lee D.; Ng S.; Agrawal A.; Winebrake J.; Hoen M.; Pandey A. Third IMO GHG Study 2014. Executive Summary and Final Report; IMO: London, U. K., 2014.

[ref7] Eyring V.; Kohler H. W.; Lauer A.; Lemper B. Emissions from International Shipping: 2. Impact of Future Technologies on Scenarios until 2050. J. Geophys. Res. 2005, 110, D17306 10.1029/2004JD005620.

[ref8] Olmer N.; Comer B.; Roy B.; Mao X.; Rutherford D. Greenhouse Gas Emissions from Global Shipping, 2013–2015; International Council on Clean Transportation: Washington DC, 2017.

[ref9] Corbett J. J.; Winebrake J. J.; Carr E. W.; Jalkanen J.-P.; Johansson L.; Prank M.; Sofiev M.; Winebrake S. G.; Karppinen A. Air Pollution and Energy Efficiency: Study on Effects of the Entry into Force of the Global 0.5% Fuel Oil Sulphur Content Limit on Human Health, 2016.

[ref10] Hassellöv I.; Turner D. R.; Lauer A.; Corbett J. J. Shipping Contributes to Ocean Acidification. Geophys. Res. Lett. 2013, 40, 2731–2736. 10.1002/grl.50521.

[ref11] Corbett J. J.; Winebrake J. J.; Green E. H.; Kasibhatla P.; Eyring V.; Lauer A. Mortality from Ship Emissions: A Global Assessment. Environ. Sci. Technol. 2007, 41, 8512–8518. 10.1021/es071686z. 18200887

[ref12] Bengtsson S.; Andersson K.; Fridell E. A Comparative Life Cycle Assessment of Marine Fuels. Proc. Inst. Mech. Eng. M J. Eng. Marit. Environ. 2011, 225, 97–110. 10.1177/1475090211402136.

[ref13] Bengtsson S.; Fridell E.; Andersson K. Environmental Assessment of Two Pathways towards the Use of Biofuels in Shipping. Energy Policy 2012, 44, 451–463. 10.1016/j.enpol.2012.02.030.

[ref14] Bengtsson S. K.; Fridell E.; Andersson K. E. Fuels for Short Sea Shipping: A Comparative Assessment with Focus on Environmental Impact. Proc. Inst. Mech. Eng. M J. Eng. Marit. Environ. 2014, 228, 44–54. 10.1177/1475090213480349.

[ref15] Bilgili L. Comparative Assessment of Alternative Marine Fuels in Life Cycle Perspective. Renew. Sustain. Energy Rev. 2021, 144, 110985 10.1016/j.rser.2021.110985.

[ref16] IMO. Adoption of the Initial IMO Strategy on Reduction of GHG Emissions from Ships and Existing IMO Activity Related to Reducing GHG Emissions in the Shipping Sector: Note by the International Maritime Organization to the UNFCCC Talanoa Dialogue; IMO, 2018.

[ref17] IMO. RESOLUTION MEPC.320(74): 2019 Guidelines for Consistent Implementation of the 0.50% Sulphur Limit Under Marpol Annex VI; IMO, 2019.

[ref18] IMO. IMO 2020 – Cutting Sulphur Oxide Emissions https://www.imo.org/en/MediaCentre/HotTopics/Pages/Sulphur-2020.aspx (accessed 2020-02-07).

[ref19] Ship&Bunker. LA/Long Beach Bunker Prices https://shipandbunker.com/prices/am/nampac/us-lax-la-long-beach#VLSFO (accessed 2023-01-11).

[ref20] Wang M.; Elgowainy A.; Lee U.; Baek K. H.; Bafana A.; Benavides P. T.; Burnham A.; Cai H.; Cappello V.; Chen P.; Gan Y.; Gracida-Alvarez U. R.; Hawkins T. R.; Iyer R. K.; Kelly J. C.; Kim T.; Kumar S.; Kwon H.; Lee K.; Liang C.; Liu X.; Lu Z.; Masum F. H.; Ng C.; Ou L.; Reddi K.; Siddique N.; Sun P.; Vyawahare P.; Xu H.; Zaimes G. Z. Greenhouse Gases, Regulated Emissions, and Energy Use in Technologies Model ® (2022 Excel); Argonne National Laboratory: Lemont, IL, 2022.

[ref21] Abdul Jameel A. G.; Han Y.; Brignoli O.; Telalović S.; Elbaz A. M.; Im H. G.; Roberts W. L.; Sarathy S. M. Heavy Fuel Oil Pyrolysis and Combustion: Kinetics and Evolved Gases Investigated by TGA-FTIR. J. Anal. Appl. Pyrolysis 2017, 127, 183–195. 10.1016/j.jaap.2017.08.008.

[ref22] Hua J.; Wu Y.; Chen H. Alternative Fuel for Sustainable Shipping across the Taiwan Strait. Transp. Res. D Transp. Environ. 2017, 52, 254–276. 10.1016/j.trd.2017.03.015.

[ref23] El-Houjeiri H.; Monfort J.; Bouchard J.; Przesmitzki S. Life Cycle Assessment of Greenhouse Gas Emissions from Marine Fuels: A Case Study of Saudi Crude Oil versus Natural Gas in Different Global Regions. J. Ind. Ecol. 2019, 23, 374–388. 10.1111/jiec.12751.

[ref24] Hoffmann J.; Asariotis R.; Assaf M.; Benamara H. Review of Maritime Transport 2018. In United Nations Conference on Trade and Development; Geneva, Switzerland, 2018.

[ref25] Kass M. D.; Abdullah Z.; Biddy M. J.; Drennan C.; Haq Z.; Hawkins T.; Jones S.; Holliday J.; Longman D. E.; Menter S.; Newes E.; Theiss T. J.; Thompson T.; Wang M. Understanding the Opportunities of Biofuels for Marine Shipping; Oak Ridge National Lab.(ORNL): Oak Ridge, TN (United States), 2018; Vol. 55.

[ref26] Xing H.; Stuart C.; Spence S.; Chen H. Alternative Fuel Options for Low Carbon Maritime Transportation: Pathways to 2050. J. Cleaner Prod. 2021, 297, 126651 10.1016/j.jclepro.2021.126651.

[ref27] Al-Enazi A.; Okonkwo E. C.; Bicer Y.; Al-Ansari T. A Review of Cleaner Alternative Fuels for Maritime Transportation. Energy Rep. 2021, 7, 1962–1985. 10.1016/j.egyr.2021.03.036.

[ref28] Chu Van T.; Ramirez J.; Rainey T.; Ristovski Z.; Brown R. J. Global Impacts of Recent IMO Regulations on Marine Fuel Oil Refining Processes and Ship Emissions. Transp. Res. D Transp. Environ. 2019, 70, 123–134. 10.1016/j.trd.2019.04.001.

[ref29] Bouman E. A.; Lindstad E.; Rialland A. I.; Strømman A. H. State-of-the-Art Technologies, Measures, and Potential for Reducing GHG Emissions from Shipping – A Review. Transp. Res. D Transp. Environ. 2017, 52, 408–421. 10.1016/j.trd.2017.03.022.

[ref30] Tan E. C. D.; Hawkins T. R.; Lee U.; Tao L.; Meyer P. A.; Wang M.; Thompson T. Biofuel Options for Marine Applications: Technoeconomic and Life-Cycle Analyses. Environ. Sci. Technol. 2021, 55, 7561–7570. 10.1021/acs.est.0c06141. 33998807

[ref31] Ampah J. D.; Yusuf A. A.; Afrane S.; Jin C.; Liu H. Reviewing Two Decades of Cleaner Alternative Marine Fuels: Towards IMO’s Decarbonization of the Maritime Transport Sector. J. Cleaner Prod. 2021, 320, 128871 10.1016/j.jclepro.2021.128871.

[ref32] Uria-Martinez R.; Corbett J. J.; Wang Z. Primer on the Cost of Marine Fuels Compliant with IMO 2020 Rule; Oak Ridge National Lab.(ORNL): Oak Ridge, 2021.

[ref33] de Fournas N.; Wei M. Techno-Economic Assessment of Renewable Methanol from Biomass Gasification and PEM Electrolysis for Decarbonization of the Maritime Sector in California. Energy Convers. Manage. 2022, 257, 115440 10.1016/j.enconman.2022.115440.

[ref34] Wang Y.; Wright L. A. A Comparative Review of Alternative Fuels for the Maritime Sector: Economic, Technology, and Policy Challenges for Clean Energy Implementation. World 2021, 2, 456–481. 10.3390/world2040029.

[ref35] Tock L.; Gassner M.; Maréchal F. Thermochemical Production of Liquid Fuels from Biomass: Thermo-Economic Modeling, Process Design and Process Integration Analysis. Biomass Bioenergy 2010, 34, 1838–1854. 10.1016/j.biombioe.2010.07.018.

[ref36] Tan E. C. D.; Harris K.; Tifft S. M.; Steward D.; Kinchin C.; Thompson T. N. Adoption of Biofuels for Marine Shipping Decarbonization: A Long-Term Price and Scalability Assessment. Biofuels, Bioprod. Biorefin. 2022, 16, 942 10.1002/bbb.2350.

[ref37] Lozano E. M.; Løkke S.; Rosendahl L. A.; Pedersen T. H. Production of Marine Biofuels from Hydrothermal Liquefaction of Sewage Sludge. Preliminary Techno-Economic Analysis and Life-Cycle GHG Emissions Assessment of Dutch Case Study. Energy Convers. Manag. X 2022, 14, 100178 10.1016/j.ecmx.2022.100178.

[ref38] Kass M. D.; Armstrong B. L.; Kaul B. C.; Connatser R. M.; Lewis S.; Keiser J. R.; Jun J.; Warrington G.; Sulejmanovic D. Stability, Combustion, and Compatibility of High-Viscosity Heavy Fuel Oil Blends with a Fast Pyrolysis Bio-Oil. Energy Fuels 2020, 34, 8403–8413. 10.1021/acs.energyfuels.0c00721.

[ref39] Kouris P. D.; van Osch D. J. G. P.; Cremers G. J. W.; Boot M. D.; Hensen E. J. M. Mild Thermolytic Solvolysis of Technical Lignins in Polar Organic Solvents to a Crude Lignin Oil. Sustain. Energy Fuels 2020, 4, 6212–6226. 10.1039/D0SE01016B.

[ref40] Maersk. Maersk Join Forces with Industry Peers and Customers to Develop LEO: Press Release by Maersk https://www.maersk.com/%7E/media_sc9/maersk/news/press-releases/files/2019/10/2019_co2_leo_press-release.pdf (accessed 2022-02-07).

[ref41] Zhang Z.; Kouris G. D.; Kouris P. D.; Hensen E. J. M.; Boot M. D.; Wu D. Investigation of the Combustion and Emissions of Lignin-derived Aromatic Oxygenates in a Marine Diesel Engine. Biofuels, Bioprod. Biorefin. 2021, 15, 1709–1724. 10.1002/bbb.2267.

[ref42] Brynolf S.; Fridell E.; Andersson K. Environmental Assessment of Marine Fuels: Liquefied Natural Gas, Liquefied Biogas, Methanol and Bio-Methanol. J. Cleaner Prod. 2014, 74, 86–95. 10.1016/j.jclepro.2014.03.052.

[ref43] Foretich A.; Zaimes G. G.; Hawkins T. R.; Newes E. Challenges and Opportunities for Alternative Fuels in the Maritime Sector. Marit. Transp. Res. 2021, 2, 100033 10.1016/j.martra.2021.100033.

[ref44] Dutta A.; Iisa K.; Mukarakate C.; Griffin M.; Tan E. C. D.; Schaidle J.; Humbird D.; Wang H.; Hartley D.; Thompson D.; Cai H.; Dutta A.; Iisa K.; Mukarakate C.; Griffin M.; Tan E. C. D.; Schaidle J.; Humbird D.; Wang H.; Hartley D.; Thompson D.; Cai H. Ex Situ Catalytic Fast Pyrolysis of Lignocellulosic Biomass to Hydrocarbon Fuels: 2019 State of Technology and Future Research, NREL/TP-51; National Renewable Energy Lab, 2018; pp. 1–43.

[ref45] Campanario F. J.; Gutiérrez Ortiz F. J. Techno-Economic Assessment of Bio-Oil Aqueous Phase-to-Liquids via Fischer-Tropsch Synthesis and Based on Supercritical Water Reforming. Energy Convers. Manage. 2017, 154, 591–602. 10.1016/j.enconman.2017.10.096.

[ref46] Li S.; Tan E. C. D.; Dutta A.; Snowden-Swan L. J.; Thorson M. R.; Ramasamy K. K.; Bartling A. W.; Brasington R.; Kass M. D.; Zaimes G. G.; Hawkins T. R. Techno-Economic Analysis of Sustainable Biofuels for Marine Transportation. Environ. Sci. Technol. 2022, 56, 17206–17214. 10.1021/acs.est.2c03960. 36409825 PMC9730900

[ref47] Ou L.; Li S.; Tao L.; Phillips S.; Hawkins T.; Singh A.; Snowden-Swan L.; Cai H. Techno-Economic Analysis and Life-Cycle Analysis of Renewable Diesel Fuels Produced with Waste Feedstocks. ACS Sustainable Chem. Eng. 2022, 10, 382–393. 10.1021/acssuschemeng.1c06561.

[ref48] Wang M.; Elgowainy A.; Lee U.; Baek K.; Bafana A.; Benavides P.; Burnham A.; Cai H.; Cappello V.; Chen P.; Gan Y.; Gracida-Alvarez U.; Hawkins T.; Iyer R.; Kelly J.; Kim T.; Kumar S.; Kwon H.; Lee K.; Liu X.; Lu Z.; Masum F.; Ng C.; Ou L.; Reddi K.; Siddique N.; Sun P.; Vyawahare P.; Xu H.; Zaimes G. Summary of Expansions and Updates in GREET® 2022; Argonne National Lab.(ANL): Argonne, IL (United States), 2022.

[ref49] Langholtz M. H.; Stokes B. J.; Eaton L. M. 2016 Billion-Ton Report: Advancing Domestic Resources for a Thriving Bioeconomy, Volume 1: Economic Availability of Feedstocks, ORNL/TM-2016/160; Oak Ridge National Lab.(ORNL): Oak Ridge, TN (United States), 2016.

[ref50] Oke D.; Dunn J. B.; Hawkins T. R. The Contribution of Biomass and Waste Resources to Decarbonizing Transportation and Related Energy and Environmental Effects. Sustain. Energy Fuels 2022, 6, 721–735. 10.1039/d1se01742j.

[ref51] Gilbert P.; Walsh C.; Traut M.; Kesieme U.; Pazouki K.; Murphy A. Assessment of Full Life-Cycle Air Emissions of Alternative Shipping Fuels. J. Cleaner Prod. 2018, 172, 855–866. 10.1016/j.jclepro.2017.10.165.

[ref52] California Air Resources Board. LCFS Credit Clearance Market: California Air Resources Board https://ww2.arb.ca.gov/resources/documents/lcfs-credit-clearance-market (accessed 2022-05-02).

[ref53] Masum F. H.; Coppola E.; Field J. L.; Geller D.; George S.; Miller J. L.; Mulvaney M. J.; Nana S.; Seepaul R.; Small I. M.; Wright D.; Dwivedi P. Supply Chain Optimization of Sustainable Aviation Fuel from Carinata in the Southeastern United States. Renew. Sustain. Energy Rev. 2023, 171, 113032 10.1016/j.rser.2022.113032.

[ref54] Friedmann S. J.; Fan Z.; Byrum Z.; Ochu E.; Bhardwaj A.; Sheerazi H. Levelized Cost of Carbon Abatement: An Improved Cost-Assessment Methodology for a Net-Zero Emissions World; Center on Global Energy Policy, Columbia University; New York, NY, 2020.

[ref55] Ullah K. M.; Masum F. H.; Field J. L.; Dwivedi P. Designing a GIS-based Supply Chain for Producing Carinata-based Sustainable Aviation Fuel in Georgia, USA. Biofuels, Bioprod. Biorefin. 2023, 1–17. 10.1002/bbb.2483.

[ref56] California Air Resources Board. LCFS Data Dashboard. https://ww2.arb.ca.gov/resources/documents/lcfs-data-dashboard (accessed 2023-01-12).

[ref57] US EIA. Prices for California’s emissions credits increase in early 2022 auction. https://www.eia.gov/todayinenergy/detail.php?id=51918#:%7E:text=In (accessed 2023-01-12).

[ref58] Trading Economics. EU Carbon Permits. https://tradingeconomics.com/commodity/carbon (accessed 2023-01-12).

[ref59] Capaz R. S.; Guida E.; Seabra J. E. A.; Osseweijer P.; Posada J. A. Mitigating Carbon Emissions through Sustainable Aviation Fuels: Costs and Potential. Biofuels, Bioprod. Biorefin. 2021, 15, 502–524. 10.1002/bbb.2168.

[ref60] Wahl J.; Kallo J. Carbon Abatement Cost of Hydrogen Based Synthetic Fuels – A General Framework Exemplarily Applied to the Maritime Sector. Int. J. Hydrogen Energy 2022, 47, 3515–3531. 10.1016/j.ijhydene.2021.11.035.

[ref61] Lee U.; Cai H.; Ou L.; Benavides P. T.; Wang Y.; Wang M. Life Cycle Analysis of Gasification and Fischer-Tropsch Conversion of Municipal Solid Waste for Transportation Fuel Production. J. Cleaner Prod. 2023, 382, 135114 10.1016/j.jclepro.2022.135114.

[ref62] Xu H.; Lee U.; Coleman A. M.; Wigmosta M. S.; Wang M. Assessment of Algal Biofuel Resource Potential in the United States with Consideration of Regional Water Stress. Algal Res. 2019, 37, 30–39. 10.1016/j.algal.2018.11.002.

[ref63] Fortier M. O. P.; Roberts G. W.; Stagg-Williams S. M.; Sturm B. S. M. Life Cycle Assessment of Bio-Jet Fuel from Hydrothermal Liquefaction of Microalgae. Appl. Energy 2014, 122, 73–82. 10.1016/j.apenergy.2014.01.077.

