# Supplementary material for: Comparing Life-Cycle Emissions of Biofuels for Marine Applications: Hydrothermal Liquefaction of Wet Wastes, Pyrolysis of Wood, Fischer–Tropsch Synthesis of Landfill Gas, and Solvolysis of Wood
Source: Environ Sci Technol. 2023 Aug 17;57(34):12701–12. doi: 10.1021/acs.est.3c00388 (PMC10469451; doi:10.1021/acs.est.3c00388)
Supplement: Supplementary file 1 — es3c00388_si_001.pdf [file es3c00388_si_001.pdf]

Supporting Information

Comparing Life-Cycle Emissions of Biofuels for Marine Applications: Hydrothermal Liquefaction of Wet Wastes, Pyrolysis of Wood, Fischer Tropsch Synthesis of Landfill Gas, and Solvolysis of Wood

Farhad H. Masum[1], George G. Zaimes[1], Eric C.D. Tan[2], Shuyun Li[3], Abhijit Dutta[2], Karthikeyan K. Ramasamy[3], and Troy R. Hawkins[1,*]
[1]Argonne National Laboratory, 9700 S Cass Avenue, Lemont, IL 60439, USA
[2]National Renewable Energy Laboratory, Golden, CO 80401, USA
[3]Pacific Northwest National Laboratory, Richland, WA 99352, USA
*Corresponding author: Troy R. Hawkins
*Email: thawkins@anl.gov

Summary: 20 pages, 15 tables, 4 figures

List of Tables

Table S1: Life cycle inventory of marine fuel from sludge and manure pathway 2
Table S2: Fuel properties and inventory for partially or fully upgrading biocrude to hydrotreated fuel 3
Table S3: Life cycle Inventory for fast pyrolysis-based pathways 4
Table S4: Life cycle Inventory for landfill gas-based pathway 5
Table S5: Life cycle Inventory for LEO-based pathway 6
Table S6: Emission parameters for feedstock 7
Table S7: Emission parameters for energy carrier and energy combustion 8
Table S8: Emission parameters for material and chemical inputs 9
Table S9: Emission parameters for catalyst inputs 10
Table S10: Emission parameters for process emissions and water use 11
Table S11: Emission parameters for displacement and counterfactual credit 12
Table S12: Emission parameters for C sequestration 13
Table S13: Emission parameters for transportation 14
Table S14: Emission parameters for fuel combustion 15
Table S15: Minimum fuel selling price* 16

List of Figures

Figure S1: Life cycle PM10 emissions of marine biofuel pathways 17
Figure S2: Life cycle NOx emissions of marine biofuel pathways 18
Figure S3: Total energy consumption of marine biofuel pathways 19
Figure S4: Total water consumption of marine biofuel pathways 20

Table S1: Life cycle inventory of marine fuel from sludge and manure pathway

	Unit	1000 MTPD Sludge HTL with NH3 removal	1000 MTPD Manure HTL with NH3 removal
Wet Waste Collection Distance*	km	47.3	115.08
Wet Waste Properties			
Moisture content	%	75	75
Ash content (dry basis)	%	15.02	12
Biocrude Properties			
Moisture content	%	4	4
Density	g/L	976.6	976.6
LHV	MJ/L	34.74	30.92
Carbon Content (wet basis)	wt%	74.92%	71.6%
Sulfur Content (wet basis)	wt%	1.11%	0.7%
Inputs			
Sludge (dry basis)	kg/hr	41579	41579
Natural gas	kg/hr	1717	2050
Electricity, kW (HTL process)	kw	1638	1671.8
Electricity, kW (at WWTP for COD)	kw	4251.62	6808.10
Dewatering polymer	kg/hr	109	109
Quicklime (CaO)	kg/hr	2155	1146
Cooling water makeup	kg/hr	967	895
Outputs			
Biocrude	kg/hr	17475	19123
Aqueous phase	kg/hr	126155	127667
HTL Solids	kg/hr	25194	17282
Solids from HTL Aqueous Treatment	kg/hr	4496	2381

* for 1000 metric ton per day (MTPD) scale

Table S2: Fuel properties and inventory for partially or fully upgrading biocrude to hydrotreated fuel

		Partially upgraded fuel from sludge	Fully upgraded fuel from sludge	Partially upgraded fuel from manure	Fully upgraded fuel from manure
Fuel Product Properties					
Fuel Density	g/L	961	963.4	961	962.9
Energy Content	MJ/L	41.36	41.70	40.81	40.88
Carbon Content	wt%	85.34%	85.65%	85.34%	84.70%
Sulfur Content	ppm	3895.06	10.65	3895.06	52.51
Inputs					
Biocrude	kg/hr	17474.65	17474.65	19210.54	19210.54
Natural gas	kg/hr	23.13	1006.79	1196.12	2213.53
Electricity, kW	kW	300.00	730.53	852.00	754.00
Cooling tower chemical	kg/hr	0.17	0.23	0.12	0.23
Boiler chemical	kg/hr	0.14	0.14	0.09	0.16
Hydrotreating catalyst, CoMo	kg/hr	1.37	1.37	1.37	1.37
Hydrotreating catalyst, NiMo	kg/hr	0	1.08	0	1.08
Hydrogen plant catalyst	kg/hr	0.10	0.16	0.11	0.17
Cooling water makeup	kg/hr	10458.29	13867.68	7520.68	14315.09
Boiler feedwater makeup	kg/hr	2905.71	4355.39	2907.53	4619.38
Outputs					
Upgraded Fuel	kg/hr	13630.33	13663.56	14535.37	14734.49
Wastewater	kg/hr	5964.97	9669.68	8328.41	8387.38

Table S3: Life cycle Inventory for fast pyrolysis-based pathways

	Unit	FP	CFP (ZSM5)	CFP (Pt/TiO2)
Fuel Properties				
Lower heating value	MJ/L	19.9	30.8	31.4
Density	g/L	1197.7	974.6	971.3
C	wt%	43.7%	75.7%	75.1%
Inputs				
Hydrotreating Catalyst (sulfided CoMo)	g/hr	0.06	0.0001	0.06
Hydrocracking Catalyst (crystalline Si-Al with rare earth metals)	g/hr	0.001	0.02	0.001
Biorefinery Resource Consumption				
Blended woody biomass (wet)	kg/hr		92592.48	
Blended woody biomass (dry) 50% forest residue + 50% clean pine	kg/hr		83333.23	
Sand makeup	kg/hr	94.36	71.08	71.94
Natural Gas	g/hr	0	0.95	0
Zeolite catalyst	kg/hr	0	103.70	0
Fixed-Bed VPU Catalyst (1% Pt/TiO2)	kg/hr	0	0	6.34
50 wt% Caustic	kg/hr	104.56	104.61	104.61
Net Water Makeup	kg/hr	26480.34	21068.25	23594.14
Boiler feed water chemicals	kg/hr		0.91	
Cooling tower chemicals	kg/hr		0.45	
No. 2 diesel fuel	kg/hr	32.16	32.16	32.16
Outputs				
Whole Oil	kg/hr	59572.45	18473.40	20017.01
Total	kg/hr	59572.45	18473.40	20017.01
Excess electricity	Kwh	6473.17	47035.00	18189.63
MEK	kg/hr	0	0	420.11
Acetone	kg/hr	0	0	2420.15
Waste Streams				
Solids purge from fluidized bed reactors	kg/hr	1727.19	1819.91	1697.51
Wastewater	kg/hr	6069.21	32990.17	7713.58

Table S4: Life cycle Inventory for landfill gas-based pathway

	Unit	Amount
Fuel properties		
Lower heating value	MJ/L	34.7
Density	g/L	789.1
C	wt%	85%
Inputs		
Biogas for Reformer / Fuel Combustor	kg/hr	106,010.8
Tar Reformer Catalyst		7.5
Hydroisomerization Catalyst		7.1
Zinc Oxide (ZnO) Catalyst		2.4
FT Synthesis Catalyst (Co based)		1.5
Hydrotreating Catalyst (sulfided CoMo or NiMo)		2.7
LO-CAT chemicals		0.0
Amine Make-Up		0.01
Boiler Feed Water Makeup		24,461.7
Boiler Chemicals		0.6
Cooling Tower Chemicals		1.4
Cooling Tower Makeup		124,788.0
No. 2 Diesel Fuel		31.4
Outputs		
Diesel	kg/hr	3,447
Jet fuel		7,499
Gasoline		7,149
Wax		2,213
Hydrogen		624
Electricity (kWh/hr)	kWh	5,844

Table S5: Life cycle Inventory for LEO-based pathway

	Unit	Amount
Fuel properties		
Lower heating value	MJ/kg	21.62
Density	g/L	1496.8
C	wt%	54.26%
Inputs		
Poplar Feedstock (wet basis, 20% moisture)	kg/hr	104166.67
Poplar Feedstock (dry basis)		83333.33
Methanol		0
Hydrogen		0
Corn Steep Liquor		735.87
Diammonium Phosphate		89.41
Sorbitol		28.25
Glucose		3471.35
Corn Steep Liquor		236.15
Ammonia		165.06
Host nutrients		96.69
Sulfur Dioxide		23.57
Caustic (as pure)		0
Boiler Chemicals		0.09
FGD Lime		45.12
Natural Gas		15950
Cooling Tower Chemicals		2.8
Makeup Water		183416.06
5 wt.% Pd/C Catalyst (RCF Reactor)		1.91
Products		
Ethanol	kg/hr	18847.5
Lignin-Ethanol Oil		25463.28
Excess Electricity	kWh/hr	23513.07
Waste Streams		
Ash	kg/hr	917.14
Carbon		82.19
$CaSO_4$		66.32
LIME		9.02
Wastewater Brine		182.99

Table S6: Emission parameters for feedstock

Emissions Factors	Unit -->	Feedstock dry VS kg	wet metric ton	dry metric ton	dry metric ton	MMBtu	dry metric ton	dry metric ton
	Material Input -->	Sludge	Swine Manure	Logging Residue	Clean Pine	Biogas	Poplar	Lignin
Total energy	MJ/unit	0.06	150.63	2666.37	3353.83	0.00	727.04	1.45
Fossil fuels	MJ/unit	0.06	149.88	2509.74	3143.08	0.00	719.65	1.44
Coal	MJ/unit	0.00	1.24	266.32	358.45	0.00	12.53	0.03
Natural gas	MJ/unit	0.01	15.75	1644.88	2082.42	0.00	189.45	0.38
Petroleum	MJ/unit	0.05	132.89	598.54	702.21	0.00	517.66	1.04
Water consumption	gal/unit	0.00	10.57	282.19	532.06	0.00	119.65	0.24
VOC	g/unit	0.00	1.44	162.86	218.23	0.00	28.37	0.06
CO	g/unit	0.01	14.83	950.12	999.24	0.00	128.47	0.26
NOx	g/unit	0.00	10.98	992.10	1261.59	0.00	194.66	0.39
PM10	g/unit	0.00	0.66	21.06	37.36	0.00	14.15	0.03
PM2.5	g/unit	0.00	0.22	17.12	31.48	0.00	12.23	0.02
SOx	g/unit	0.00	0.69	44.67	106.04	0.00	25.82	0.05
BC	g/unit	0.00	0.03	6.21	9.37	0.00	6.77	0.01
OC	g/unit	0.00	0.05	5.33	9.67	0.00	2.29	0.00
CH4	g/unit	0.01	13.53	812.40	1014.70	0.00	115.42	0.23
N_2O	g/unit	0.00	0.04	2.82	92.89	0.00	51.92	0.10
CO_2	g/unit	4.57	11110.35	159789.42	205816.00	0.00	56297.02	112.59
CO_2 Sequestration	g/unit	0.00	0.00	0.00	0.00	0.00	0.00	0.00
Biogenic CH_4	g/unit	0.00	0.00	0.00	0.00	0.00	0.00	0.00
Biogenic CO_2	g/unit	0.00	0.00	0.00	0.00	0.00	0.00	0.00
CO_2 (w/ C in VOC & CO)	g/unit	4.58	11138.16	161790.04	208066.37	0.00	56587.31	113.17
GHGs	g/unit	4.75	11552.70	186770.71	263663.82	0.00	74200.69	148.40

Table S7: Emission parameters for energy carrier and energy combustion

Parameter		Energy Carrier			Energy Combustion		
		Natural gas	Electricity	Diesel	Natural gas combustion, SMR	Natural gas combustion, Utility/ Industrial Boiler	Diesel Combustion, Industrial Boiler
Total energy	MJ/MJ	0.11	2.07	0.19	1.00	1.00	1.00
Fossil fuels	MJ/MJ	0.11	1.64	0.18	1.00	1.00	1.00
Coal	MJ/MJ	0.00	0.73	0.01	0.00	0.00	0.00
Natural gas	MJ/MJ	0.11	0.89	0.12	1.00	1.00	0.00
Petroleum	MJ/MJ	0.00	0.02	0.05	0.00	0.00	1.00
Water consumption	L/MJ	0.01	0.62	0.08	0.00	0.00	0.00
VOC	g/MJ	0.01	0.01	0.01	0.00	0.00	0.00
CO	g/MJ	0.03	0.05	0.01	0.00	0.02	0.02
NOx	g/MJ	0.04	0.09	0.02	0.01	0.03	0.08
PM10	g/MJ	0.00	0.01	0.00	0.00	0.00	0.01
PM2.5	g/MJ	0.00	0.01	0.00	0.00	0.00	0.00
SOx	g/MJ	0.01	0.08	0.00	0.00	0.00	0.00
BC	g/MJ	0.00	0.00	0.00	0.00	0.00	0.00
OC	g/MJ	0.00	0.00	0.00	0.00	0.00	0.00
CH4	g/MJ	0.21	0.26	0.11	0.00	0.00	0.00
N_2O	g/MJ	0.00	0.00	0.00	0.00	0.00	0.00
CO_2	g/MJ	6.27	121.13	12.50	56.30	56.27	74.09
CO_2 Sequestration	g/MJ	0.00	0.00	0.00	0.00	0.00	0.00
Biogenic CH_4	g/MJ	0.00	0.00	0.00	0.00	0.00	0.00
Biogenic CO_2	g/MJ	0.00	0.00	0.00	0.00	0.00	0.00
CO_2 (w/ C in VOC & CO)	g/MJ	6.35	121.26	12.54	56.31	56.31	74.12
GHGs	g/MJ	13.02	129.58	15.76	56.53	56.53	74.22

Table S8: Emission parameters for material and chemical inputs

Parameter	Unit →	MJ	metric ton										
		Ethanol	Sand	Polymer	Quicklime (CaO)	Sulfuric Acid, 93%	Ammonia	Corn Steep Liquor	Diammonium Phosphate (DAP)	Glucose	Caustic	FGD Lime	Caustic (50% wt.)
Total energy	MJ/unit	0.9	0.4	21.1	5.0	0.0	0.0	0.0	0.0	0.6	37.7	101.0	24.8
Fossil fuels		0.6	0.3	18.6	4.9	0.0	0.0	0.0	0.0	0.5	37.6	12.7	23.6
Coal		0.0	0.1	4.2	4.1	0.0	0.0	0.0	0.0	0.1	0.2	0.6	2.0
Natural gas		0.5	0.2	14.3	0.5	0.0	0.0	0.0	0.0	0.1	36.3	8.6	16.4
Petroleum		0.1	0.0	0.1	0.3	0.0	0.0	0.0	0.0	0.3	1.1	3.5	5.2
Water consumption	L/unit	1.5	0.1	3.7	4.7	0.0	0.0	0.0	0.0	0.3	1.7	124.8	36.8
VOC	g/unit	0.1	0.0	0.2	0.1	0.0	0.0	0.0	0.0	0.0	5.1	0.7	1.5
CO	g/unit	0.0	0.0	0.7	0.5	0.0	0.0	0.0	0.0	0.0	5.3	1.6	2.1
NOx	g/unit	0.1	0.0	1.1	0.3	0.0	0.0	0.0	0.0	0.3	2.1	3.8	4.9
PM10	g/unit	0.0	0.0	0.1	0.1	0.0	0.0	0.0	0.0	0.0	0.1	0.2	1.1
PM2.5	g/unit	0.0	0.0	0.1	0.1	0.0	0.0	0.0	0.0	0.0	0.1	0.2	0.8
SOx	g/unit	0.0	0.0	0.6	0.1	0.0	0.0	0.0	0.0	2.0	0.7	1.1	11.1
BC	g/unit	0.0	0.0	0.0	0.0	0.0	0.0	0.0	0.0	0.0	0.0	0.0	0.0
OC	g/unit	0.0	0.0	0.0	0.0	0.0	0.0	0.0	0.0	0.0	0.0	0.0	0.1
CH4	g/unit	0.1	0.0	3.2	0.7	0.0	0.0	0.0	0.0	0.1	20.9	3.5	6.8
N_2O	g/unit	0.0	0.0	0.0	0.0	0.0	0.0	0.0	0.0	0.0	0.0	2.7	0.0
CO_2	g/unit	41.4	22.5	1213.5	1262.7	0.0	0.0	0.0	0.0	38.8	2184.5	877.5	1520.3
CO_2 Sequestration	g/unit	0.0	0.0	0.0	0.0	0.0	0.0	0.0	0.0	0.0	0.0	0.0	0.0
Biogenic CH_4	g/unit	0.0	0.0	0.0	0.0	0.0	0.0	0.0	0.0	0.0	0.0	0.0	0.0
Biogenic CO_2	g/unit	0.0	0.0	0.0	0.0	0.0	0.0	0.0	0.0	0.0	0.0	0.0	0.0
CO2 (w/ C in VOC & CO)	g/unit	41.6	22.6	1215.3	1263.7	0.0	0.0	0.0	0.0	38.9	2208.7	882.4	1528.2
GHGs	g/unit	55.4	24.1	1320.0	1284.4	0.0	0.0	0.0	0.0	41.0	2842.9	1724.2	1741.2

Table S9: Emission parameters for catalyst inputs

Parameter	Unit	Mo/Co/Al_2O_3 catalyst	Pt/ Gamma Al_2O_3	ZSM-5	Tar reformer catalyst	Zinc Oxide (ZnO) Catalyst
Total energy	MJ/kg	133.1	1349.2	209.6	72.8	83.7
Fossil fuels	MJ/kg	126.8	1292.3	203.7	68.7	76.8
Coal	MJ/kg	11.4	1187.6	18.0	8.4	30.3
Natural gas	MJ/kg	68.3	18.6	178.4	57.5	17.6
Petroleum	MJ/kg	47.2	86.1	7.3	2.8	28.9
Water consumption	L/kg	35.5	952.6	21.4	25.1	22.8
VOC	g/kg	1.9	10.6	2.8	1.3	0.6
CO	g/kg	10.7	47.3	9.7	3.6	2.2
NOx	g/kg	11.0	99.8	12.9	5.8	5.7
PM10	g/kg	4.2	18.3	1.1	0.4	2.6
PM2.5	g/kg	0.8	8.0	0.9	0.3	1.3
SOx	g/kg	7.5	126.3	6.8	4.6	4.5
BC	g/kg	0.1	0.4	0.1	0.0	0.0
OC	g/kg	0.1	0.7	0.3	0.1	0.1
CH4	g/kg	19.1	190.8	36.5	13.9	9.6
N_2O	g/kg	0.5	2.6	0.3	1.9	0.1
CO_2	g/kg	8670.8	120019.6	11027.8	5327.5	6042.5
CO_2 Sequestration	g/kg	0.0	0.0	0.0	0.0	0.0
Biogenic CH_4	g/kg	0.0	0.0	0.0	0.0	0.0
Biogenic CO_2	g/kg	0.0	0.0	0.0	0.0	0.0
CO_2 (w/ C in VOC & CO)	g/kg	8693.7	120127.2	11051.9	5337.3	6047.7
GHGs	g/kg	9404.7	126514.1	12216.5	6262.9	6358.0

Table S10: Emission parameters for process emissions and water use

	Unit -->	MJ	MJ	MJ	MJ	MJ	MJ	MJ	MJ	MJ	MJ	MJ
Total energy	MJ/unit	Pathway 1	Pathway 2	Pathway 3	Pathway 4	Pathway 5	Pathway 6	Pathway 7	Pathway 8	Pathway 9	Pathway 10	Pathway 11
Fossil fuels	MJ/unit	0.00	0.00	0.00	0.00	0.00	0.00	0.00	0.00	0.00	0.00	0.00
Coal	MJ/unit	0.00	0.00	0.00	0.00	0.00	0.00	0.00	0.00	0.00	0.00	0.00
Natural gas	MJ/unit	0.00	0.00	0.00	0.00	0.00	0.00	0.00	0.00	0.00	0.00	0.00
Petroleum	MJ/unit	0.00	0.00	0.00	0.00	0.00	0.00	0.00	0.00	0.00	0.00	0.00
Water consumption	L/unit	0.00	0.00	0.00	0.00	0.00	0.00	0.00	0.00	0.00	0.00	0.00
VOC	g/unit	0.00	0.02	0.03	0.00	0.02	0.03	0.03	0.04	0.04	0.99	0.33
CO	g/unit	0.00	0.00	0.00	0.00	0.00	0.00	0.00	0.00	0.00	0.00	0.00
NOx	g/unit	0.00	0.00	0.00	0.00	0.00	0.00	0.00	0.00	0.00	0.00	0.00
PM10	g/unit	0.00	0.00	0.00	0.00	0.00	0.00	0.01	0.01	0.01	0.00	0.00
PM2.5	g/unit	0.00	0.00	0.00	0.00	0.00	0.00	0.00	0.00	0.00	0.00	0.00
SOx	g/unit	0.00	0.00	0.00	0.00	0.00	0.00	0.00	0.00	0.00	0.00	0.00
BC	g/unit	0.00	0.00	0.00	0.00	0.00	0.00	0.04	0.07	0.06	0.00	0.00
OC	g/unit	0.00	0.00	0.00	0.00	0.00	0.00	0.00	0.00	0.00	0.00	0.00
CH_4	g/unit	0.00	0.00	0.00	0.00	0.00	0.00	0.00	0.00	0.00	0.00	0.00
N_2O	g/unit	0.00	0.00	0.00	0.00	0.00	0.00	0.00	0.00	0.00	0.00	0.00
CO_2	g/unit	0.00	0.00	0.00	0.00	0.00	0.00	0.00	0.00	0.00	0.00	0.00
CO_2 Sequestration	g/unit	0.00	0.00	0.00	0.00	0.00	0.00	0.00	0.00	0.00	0.00	0.00
Biogenic CH_4	g/unit	0.00	0.00	0.00	0.00	0.00	0.00	0.00	0.00	0.00	0.00	0.00
Biogenic CO_2	g/unit	0.00	0.00	0.00	0.00	0.00	0.00	0.00	0.00	0.00	0.00	0.00
CO_2 (w/ C in VOC & CO)	g/unit	0.00	0.00	0.00	0.00	0.00	0.00	58.90	184.42	142.91	139.90	0.00
GHGs	g/unit	0.00	0.00	0.00	0.00	0.00	0.00	0.00	0.00	0.00	0.00	0.00

Table S11: Emission parameters for displacement and counterfactual credit

		Displacement			Counterfactual		
Parameter	Unit -->	MJ	kg	kg	kg VS	wet kg	MJ
Total energy	btu/unit	Electricity	Acetone	MEK	CF Sludge Waste	CF Swine Manure	CF LFG Flaring
Fossil fuels	btu/unit	2.072	65.463	43.451	11.445	-0.880	1.000
Coal	btu/unit	1.644	64.702	42.991	1.614	-0.995	0.000
Natural gas	btu/unit	0.729	2.940	0.773	0.716	-0.046	0.000
Petroleum	btu/unit	0.893	35.032	11.465	0.877	-0.838	0.000
Water consumption	gal/unit	0.022	26.733	30.753	0.021	-0.111	0.000
VOC	g/unit	0.621	0.000	153.792	0.610	-0.658	0.000
CO	g/unit	0.014	0.401	0.320	0.121	-0.092	0.022
NOx	g/unit	0.051	1.766	0.777	0.339	-0.097	0.039
PM10	g/unit	0.093	6.208	1.114	0.358	-0.135	0.015
PM2.5	g/unit	0.014	0.798	0.087	0.051	-0.028	0.005
SOx	g/unit	0.008	0.161	0.075	0.045	-0.022	0.005
BC	g/unit	0.082	6.870	0.324	0.083	-0.193	0.000
OC	g/unit	0.000	0.000	0.010	0.022	0.000	0.005
CH_4	g/unit	0.002	0.000	0.021	0.010	-0.003	0.000
N_2O	g/unit	0.257	10.377	4.668	19.080	10.507	0.030
CO_2	g/unit	0.002	0.013	0.023	0.009	0.093	0.001
CO_2 Sequestration	g/unit	121.132	2,231.078	925.608	-107.874	-88.136	0.000
Biogenic CH_4	g/unit	0.000	0.000	0.000	0.000	0.000	0.000
Biogenic CO_2	g/unit	0.000	0.000	0.000	18.828	10.921	4.639
CO_2 (w/ C in VOC & CO)	g/unit	0.000	0.000	0.000	0.000	0.000	139.896
GHGs	g/unit	121.257	2,235.104	927.827	-106.963	-88.577	0.132
Emissions Factors	Unit -->	129.583	2,547.954	1,073.064	412.190	219.961	-11.448

Table S12: Emission parameters for C sequestration

Parameter	Unit	Sludge solids	Sludge solids from aqueous treatment	Manure solids	Manure solids from aqueous treatment
Total energy	btu/kg	0.000	0.000	0.000	0.000
Fossil fuels	btu/kg	0.000	0.000	0.000	0.000
Coal	btu/kg	0.000	0.000	0.000	0.000
Natural gas	btu/kg	0.000	0.000	0.000	0.000
Petroleum	btu/kg	0.000	0.000	0.000	0.000
Water consumption	gal/kg	0.000	0.000	0.000	0.000
VOC	g/kg	0.000	0.000	0.000	0.000
CO	g/kg	0.000	0.000	0.000	0.000
NOx	g/kg	0.000	0.000	0.000	0.000
PM10	g/kg	0.000	0.000	0.000	0.000
PM2.5	g/kg	0.000	0.000	0.000	0.000
SOx	g/kg	0.000	0.000	0.000	0.000
BC	g/kg	0.000	0.000	0.000	0.000
OC	g/kg	0.000	0.000	0.000	0.000
CH_4	g/kg	0.000	0.000	0.000	0.000
N_2O	g/kg	0.000	0.000	0.000	0.000
CO_2	g/kg	0.000	0.000	0.000	0.000
CO_2 Sequestration	g/kg	-224.987	-175.120	-340.267	-313.867
Biogenic CH_4	g/kg	0.000	0.000	0.000	0.000
Biogenic CO_2	g/kg	0.000	0.000	0.000	0.000
CO_2 (w/ C in VOC & CO)	g/kg	0.000	0.000	0.000	0.000
GHGs	g/kg	-224.987	-175.120	-340.267	-313.867

Table S13: Emission parameters for transportation

		T&D	T&D-mass
Emissions Factors	Unit -->	MJ	tonne-100km
Total energy	MJ/unit	0.03	130.894
Fossil fuels	MJ/unit	0.03	130.239
Coal	MJ/unit	0.00	1.080
Natural gas	MJ/unit	0.00	13.684
Petroleum	MJ/unit	0.03	115.474
Water consumption	L/unit	0.00	9.181
VOC	g/unit	0.00	1.255
CO	g/unit	0.00	12.889
NOx	g/unit	0.04	9.541
PM10	g/unit	0.00	0.571
PM2.5	g/unit	0.00	0.189
SOx	g/unit	0.03	0.603
BC	g/unit	0.00	0.027
OC	g/unit	0.00	0.045
CH4	g/unit	0.00	11.759
N_2O	g/unit	0.00	0.036
CO_2	g/unit	2.28	9,654.461
CO_2 Sequestration	g/unit	0.00	0.000
Biogenic CH_4	g/unit	0.00	0.000
Biogenic CO_2	g/unit	0.00	0.000
CO_2 (w/ C in VOC & CO)	g/unit	2.30	9,678.627
GHGs	g/unit	2.39	10,038.839

Table S14: Emission parameters for fuel combustion

Parameter	Unit	Pathway 1	Pathway 2	Pathway 3	Pathway 4	Pathway 5	Pathway 6	Pathway 7	Pathway 8	Pathway 9	Pathway 10	Pathway 11
Total energy	MJ/MJ	1.000	1.000	1.000	1.000	1.000	1.000	1.000	1.000	1.000	1.000	1.000
Fossil fuels	MJ/MJ	0.000	0.000	0.000	0.000	0.000	0.000	0.000	0.000	0.000	0.000	0.000
Coal	MJ/MJ	0.000	0.000	0.000	0.000	0.000	0.000	0.000	0.000	0.000	0.000	0.000
Natural gas	MJ/MJ	0.000	0.000	0.000	0.000	0.000	0.000	0.000	0.000	0.000	0.000	0.000
Petroleum	MJ/MJ	0.000	0.000	0.000	0.000	0.000	0.000	0.000	0.000	0.000	0.000	0.000
Water consumption	L/MJ	0.000	0.000	0.000	0.000	0.000	0.000	0.000	0.000	0.000	0.000	0.000
VOC	g/MJ	0.057	0.057	0.057	0.057	0.057	0.057	0.057	0.057	0.057	0.057	0.057
CO	g/MJ	0.125	0.125	0.125	0.125	0.125	0.125	0.125	0.125	0.125	0.125	0.125
NOx	g/MJ	1.197	1.197	1.197	1.197	1.197	1.197	1.197	1.197	1.197	1.197	1.197
PM10	g/MJ	0.018	0.018	0.018	0.018	0.018	0.018	0.018	0.018	0.018	0.018	0.018
PM2.5	g/MJ	0.002	0.002	0.002	0.002	0.002	0.002	0.002	0.002	0.002	0.002	0.002
SOx	g/MJ	0.610	0.177	0.000	0.430	0.109	0.002	0.000	0.000	0.000	0.000	0.000
BC	g/MJ	0.000	0.000	0.000	0.000	0.000	0.000	0.000	0.000	0.000	0.000	0.000
OC	g/MJ	0.000	0.000	0.000	0.000	0.000	0.000	0.000	0.000	0.000	0.000	0.000
CH4	g/MJ	0.001	0.001	0.001	0.001	0.001	0.001	0.001	0.001	0.001	0.001	0.001
N2O	g/MJ	0.004	0.004	0.004	0.004	0.004	0.004	0.004	0.004	0.004	0.004	0.004
CO2	g/MJ	0.000	0.000	0.000	0.000	0.000	0.000	0.000	0.000	0.000	0.000	0.000
CO2 Sequestration	g/MJ	0.000	0.000	0.000	0.000	0.000	0.000	0.000	0.000	0.000	0.000	0.000
Biogenic CH_4	g/MJ	0.000	0.000	0.000	0.000	0.000	0.000	0.000	0.000	0.000	0.000	0.000
Biogenic CO_2	g/MJ	77.227	72.700	72.376	82.931	72.844	73.151	96.209	87.871	85.119	70.976	92.173
CO_2 (w/ C in VOC & CO)	g/MJ	0.375	0.375	0.375	0.375	0.375	0.375	0.375	0.375	0.375	0.375	0.375
GHGs	g/MJ	1.467	1.467	1.467	1.467	1.467	1.467	1.467	1.467	1.467	1.467	1.467

Table S15: Minimum fuel selling price*

			Minimum Fuel Selling Price			
Feedstock	Conversion & Upgrading	Primary Fuel	USD2016/MJ	USD2016 [gallon LSFO equivalent]$^{-1}$	USD2016 [tonne LSFO equivalent]$^{-1}$	USD2016 [gallon gasoline equivalent]$^{-1}$
Sludge	HTL	Biocrude	0.003	0.39	102.61	0.31
	HTL & Partial Hydrotreating	Partially upgraded fuel	0.006	0.92	244.70	0.73
	HTL & Full Hydrotreating	Fully upgraded fuel	0.009	1.26	335.47	1.01
Manure	HTL	Biocrude	0.006	0.86	228.91	0.69
	HTL & Partial Hydrotreating	Partially upgraded fuel	0.010	1.42	378.88	1.14
	HTL & Full Hydrotreating	Fully upgraded fuel	0.012	1.73	461.76	1.38
Woody Biomass	FP & Hydrotreating	FP Bio-Oil	0.025	3.65	972.81	2.92
	CFP [ZSM5] & Hydrotreating	CFP Bio-Oil	0.029	4.36	1162.05	3.49
	CFP [Pt/TiO_2] & Hydrotreating	CFP Bio-Oil	0.019	2.84	756.93	2.27
Landfill Gas (LFG)	FT Synthesis	FT-Diesel	0.025	3.76	1002.13	3.01
Poplar	Lignin solvolysis [Integrated]	Lignin Ethanol Oil	0.030	4.41	1176.13	3.53

* Findings from the technoeconomic study conducted concurrently with this research. MFSP values incorporate assumptions about the value of coproducts from the marine fuel production pathways. Results presented in gallons VLSFO equivalent are converted on the basis of an assumed lower heating value for VLSFO of 140,353 BTU/gallon and density of 3,752 grams/gallon based on the GREET fuel specifications for residual fuel.
Note: HTL = hydrothermal liquefaction, FP = Fast pyrolysis, CFP = Catalytic fast pyrolysis, FT = Fischer-Tropsch.

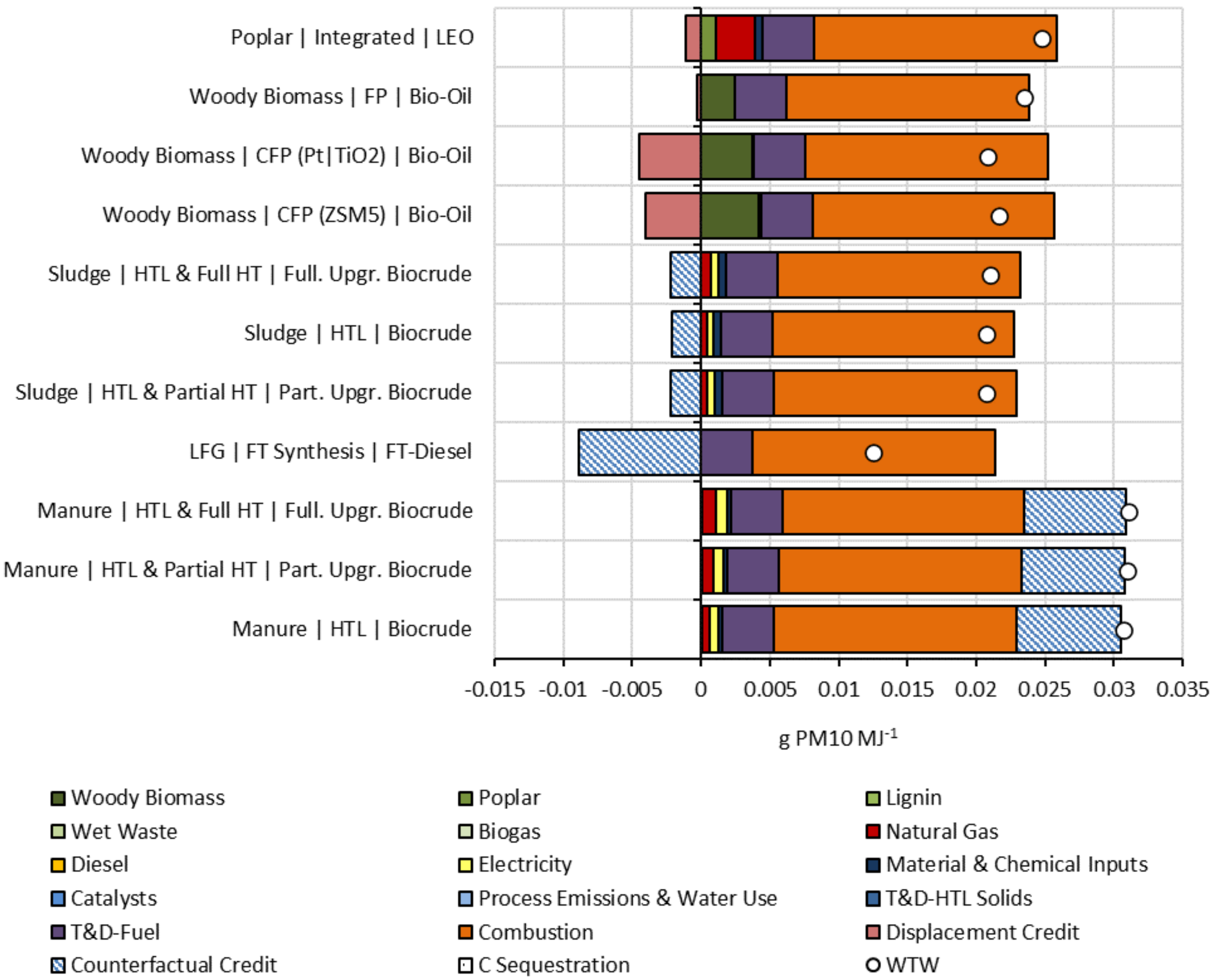

Figure S1: Life cycle PM10 emissions of marine biofuel pathways

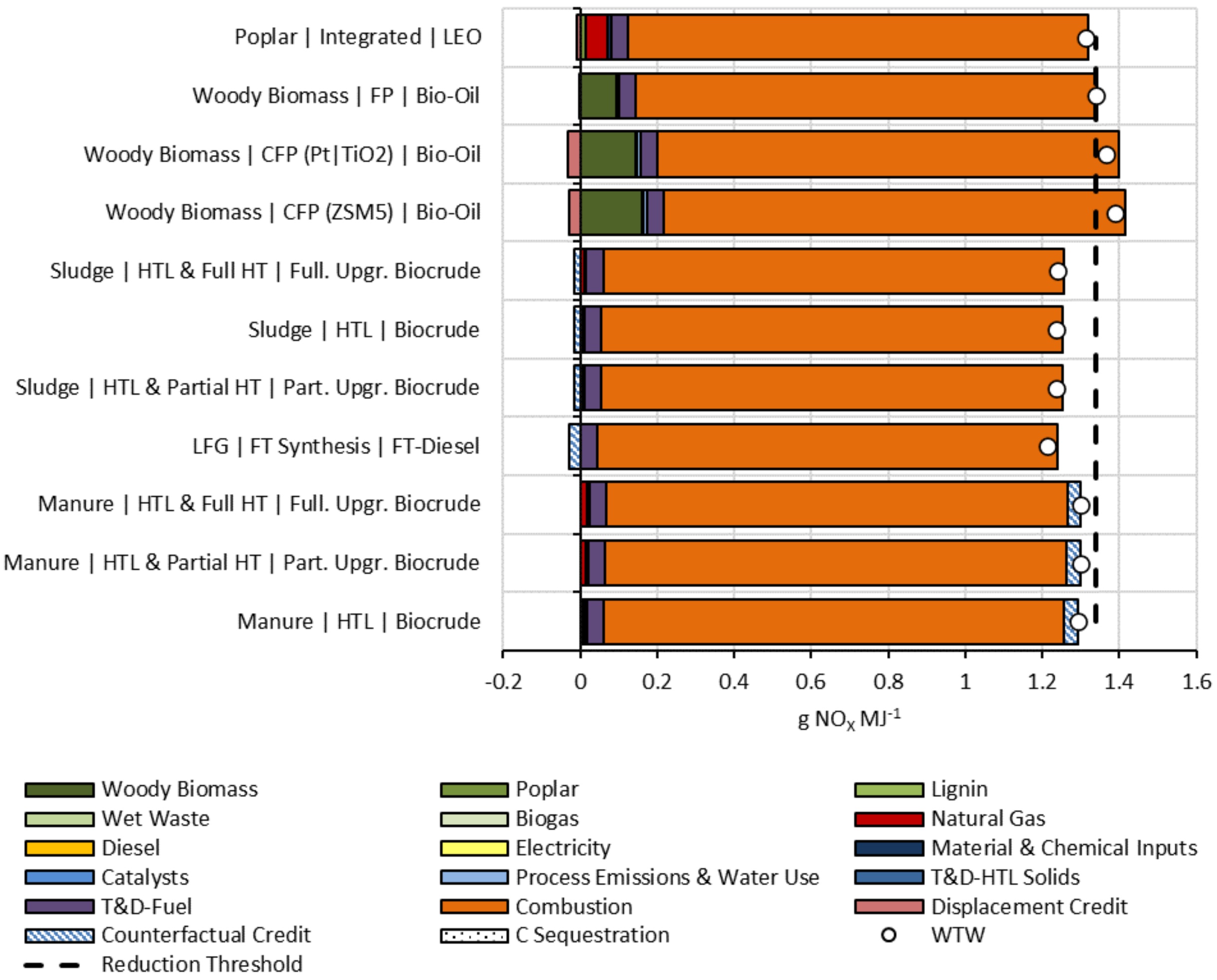

Figure S2: Life cycle NOx emissions of marine biofuel pathways

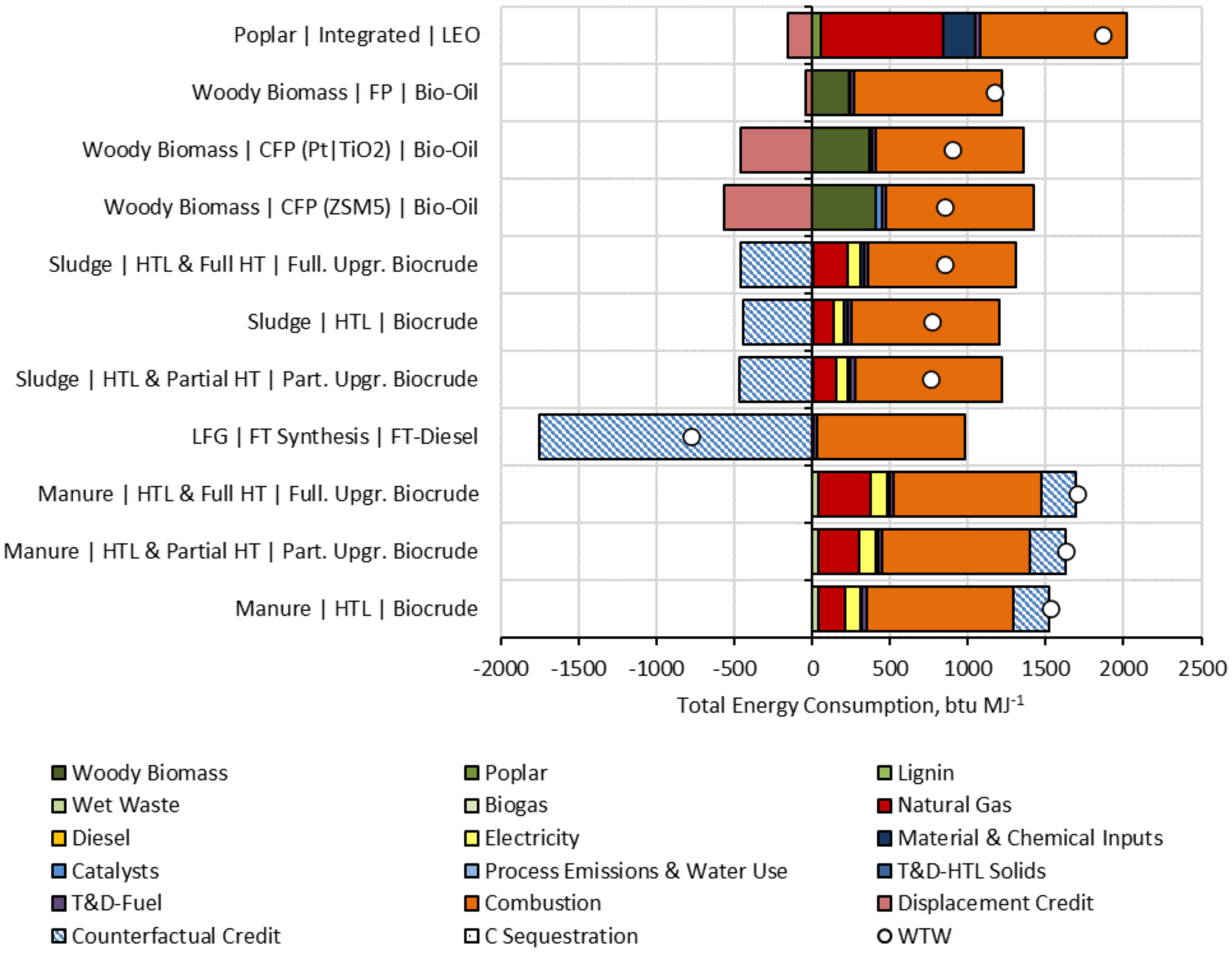

Figure S3: Total energy consumption of marine biofuel pathways

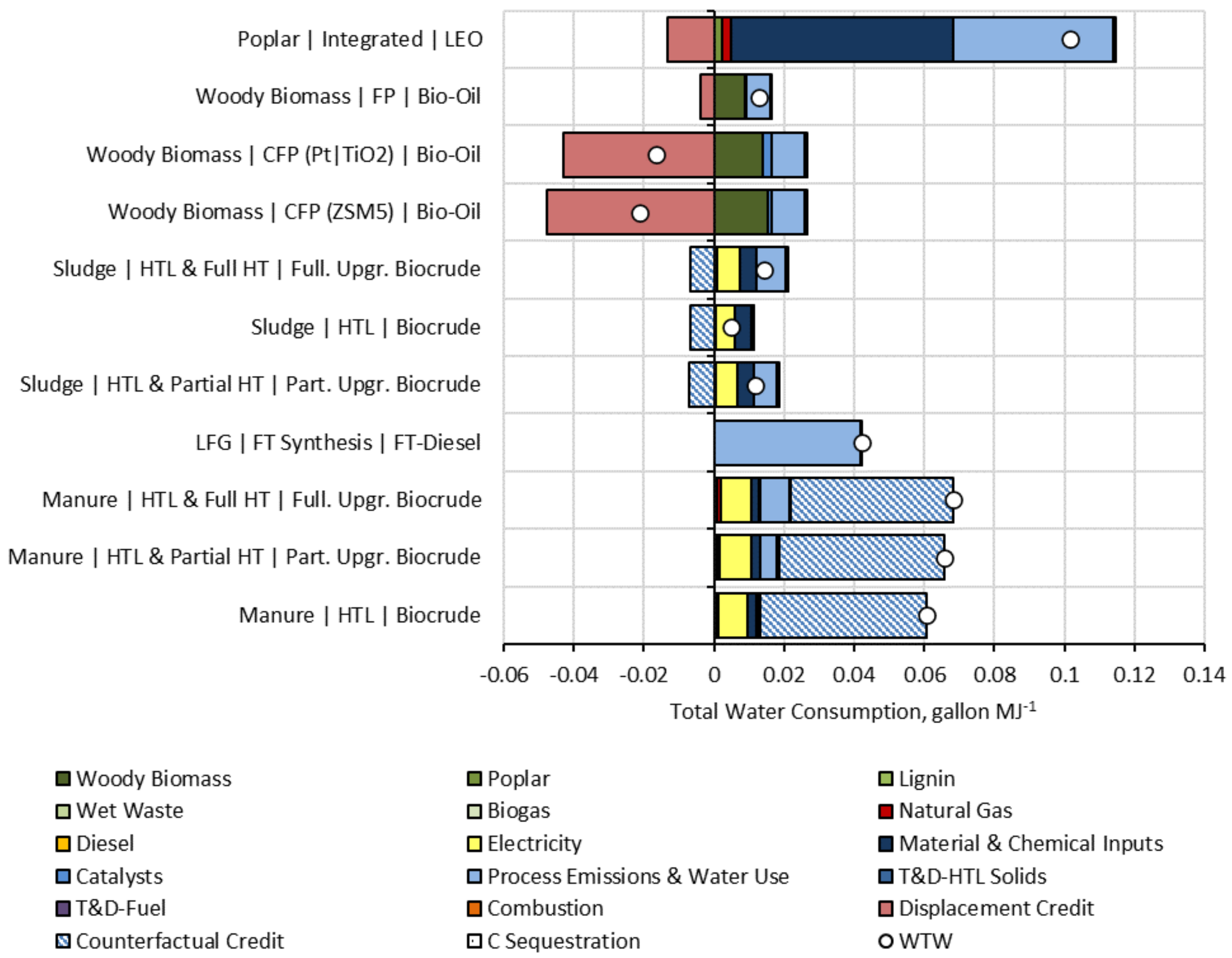

Figure S4: Total water consumption of marine biofuel pathways
